# Adaptive nitrogen-containing buckybowl: a versatile receptor for curved and planar aromatic molecules†

**DOI:** 10.1039/d5sc00988j

**Published:** 2025-03-25

**Authors:** Xu-Lang Chen, Si-Qian Yu, Zi-You Zheng, Zhao-Yi Cheng, An-Na Chen, Jia-Qi Liang, Xin Sun, Chunyang Zheng, Xiaohuan Huang, Han-Yuan Gong

**Affiliations:** a College of Chemistry and Chemical Engineering, Hubei Key Laboratory of Pollutant Analysis and Reuse Technology, Hubei Normal University Huangshi 435002 P. R. China xulangchen@hbnu.edu.cn cyzheng@hbnu.edu.cn xhuang@hbnu.edu.cn; b College of Chemistry, Beijing Normal University No. 19, Xin Jie Kou Wai St, Hai Dian District Beijing 100875 P. R. China hanyuangong@bnu.edu.cn; c College of Materials Chemistry & Chemical Engineering, Chengdu University of Technology 1#, Dongsanlu, Erxianqiao Chengdu 610059 P. R. China

## Abstract

Bowl-shaped polycyclic aromatic hydrocarbons (PAHs), or buckybowls, are renowned for their unique structures and physicochemical properties, making them promising fragments for functional materials. While well-known examples like corannulene and sumanene demonstrate their potential, synthetic challenges have limited the development of other fullerene fragments. Recent advancements, particularly the incorporation of heteroatoms, have expanded the structural diversity of buckybowls. In this study, we report the synthesis of a novel nitrogen-containing buckybowl (1) using the core-periphery strategy that connects two half-bowls *via* a single carbon–carbon bond, followed by peripheral stitching. This molecule features two nitrogen atoms within its bowl-shaped framework, representing a significant advancement in structural diversity. Compound 1 exhibits intense red emission with high color purity and quantum yield in solution. Additionally, it possesses adaptive curvature adjustment and shows excellent binding affinity to the curved PAH corannulene, the spherical PAH C_60_, as well as the planar PAH pyrene. These versatile assembly capabilities highlight its potential applications in supramolecular chemistry and materials science, paving the way for advancements in molecular electronics and photonics.

## Abstract 2

This study presents a novel nitrogen-containing buckybowl (1), synthesized by connecting two half-bowls *via* a carbon–carbon bond. Compound 1 shows red emission, adaptive curvature, and binding to corannulene, C_60_, and pyrene.

## Graphical abstract

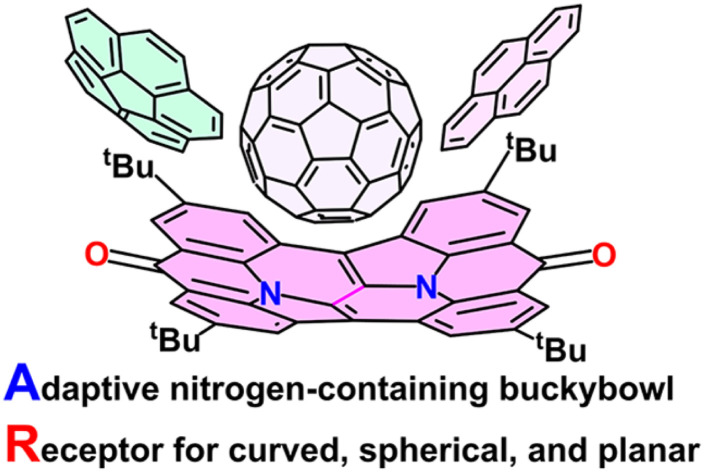

## Introduction

Bowl-shaped polycyclic aromatic hydrocarbons (PAHs), or buckybowls, are unique fullerene fragments with significant potential for functional materials.^1^ While corannulene^2^ and sumanene,^3^ fragments of C_60_, are well-studied, other fullerene fragments have been limited by synthetic challenges. Recent advances, including heteroatom incorporation, have expanded buckybowl diversity.^4^ Nitrogen-containing buckybowls,^5^ such as azasumanene,^6^ azabenzocorannulene,^7^ and bowl-shaped PAHs with nitrogen-embedded adjacent pentagons,^8^ exhibit unique structures and properties, with applications in cellular imaging,^9^ fluorescent probes,^10^ and organic light-emitting diodes (OLEDs).^11^ These molecules also form stable composites with fullerenes,^7*b*,12^ yet their assembly with other curved PAHs and planar PAHs remains underexplored, offering opportunities for creating advanced materials and devices beyond planar analogues.

**Fig. 1:**
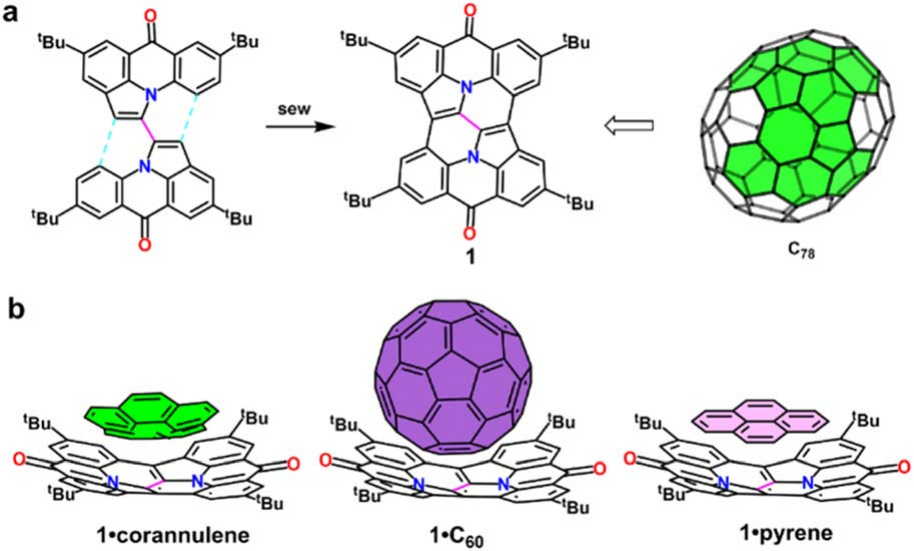
(a) The nitrogen-containing buckybowl 1, synthesized using a core-periphery strategy featuring a single-bond core, can be considered as an aza-analogue of a C_78_ fullerene fragment. (b) Schematic representation of the interactions between 1 and corannulene, C_60_, or pyrene.

Synthetic strategies for nitrogen-containing buckybowls are broadly classified into core-periphery and periphery-core approaches.^12*e*^ The core-periphery strategy, which involves constructing a central core followed by peripheral stitching, has been extensively employed to synthesize diverse structures.^6*a*,7*a*–*c*^ In this study, we present a novel method that connects two half-bowls *via* a single carbon–carbon bond, subsequently followed by peripheral stitching, resulting in the synthesis of a novel nitrogen-containing buckybowl (1). The all-carbon analog of this nitrogen-containing buckybowl can be considered a fragment derived from fullerene C_78_ (Fig. 1a). Compound 1, featuring two nitrogen atoms within its framework and a single carbon–carbon bond at its core, exhibits intense red emission in solution, achieving a maximum fluorescence quantum yield of 0.57 and high color purity, with a full width at half maximum (FWHM) of 35 nm. Additionally, the adaptive curvature adjustment property of compound 1 enables it to bind effectively to PAHs with curved surfaces, such as corannulene and C_60_, as well as planar PAHs like pyrene (Fig. 1b). This unique self-assembly capability positions compound 1 as a promising candidate for applications in supramolecular chemistry, molecular electronics, and photonics.

## Results and discussion

### Synthesis and molecular characterization

**Scheme 1:**
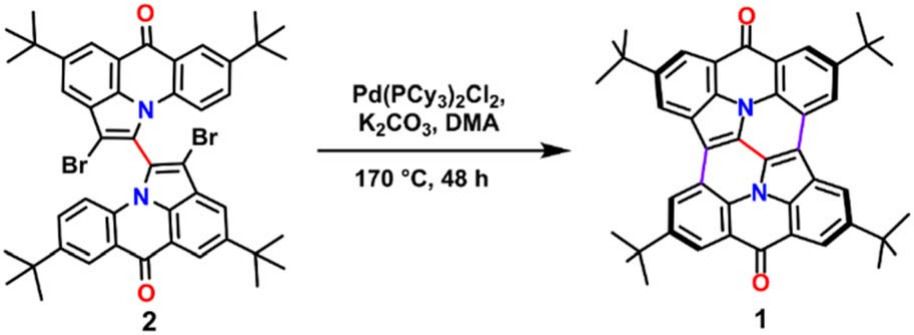
Synthetic route for the preparation of nitrogen-containing buckybowl 1.

The synthetic route began with 2,2′-dibromo-4,4′,8,8′-tetra-*tert*-butyl-6*H*,6′*H*-[1,1′-bipyrrolo[3,2,1-*de*]acridine]-6,6′-dione (2)^13^ as the starting material. Using an intramolecular palladium-catalyzed coupling reaction, we successfully generated compound 1 with a modest yield of 4% (Scheme 1). Despite the limited yield, the reaction demonstrates the feasibility of constructing this complex structure under mild conditions. Compound 1 was comprehensively characterized. Proton nuclear magnetic resonance (^1^H NMR) spectroscopy in CDCl_3_ solvent revealed six singlet signals (see Fig. S1†), while ^13^C NMR spectroscopy displayed nineteen distinct signals (see Fig. S2†). These spectral data indicate that the palladium-catalyzed intramolecular direct arylation effectively removes two hydrogen and two bromine atoms from precursor 2, facilitating the formation of two new carbon–carbon bonds. Furthermore, matrix-assisted laser desorption/ionization time-of-flight high-resolution mass spectrometry (MALDI-TOF HRMS) analysis showed an isotope pattern consistent with the simulated data (*m*/*z* calculated for [M]^+^: 656.3397; found: 656.3398; see Fig. S3†), thereby confirming the molecular integrity of compound 1. Additionally, the bowl-shaped conformation of compound 1 was corroborated by single-crystal X-ray diffraction analysis of its complex (see infra, Fig. 5).

**Fig. 2:**
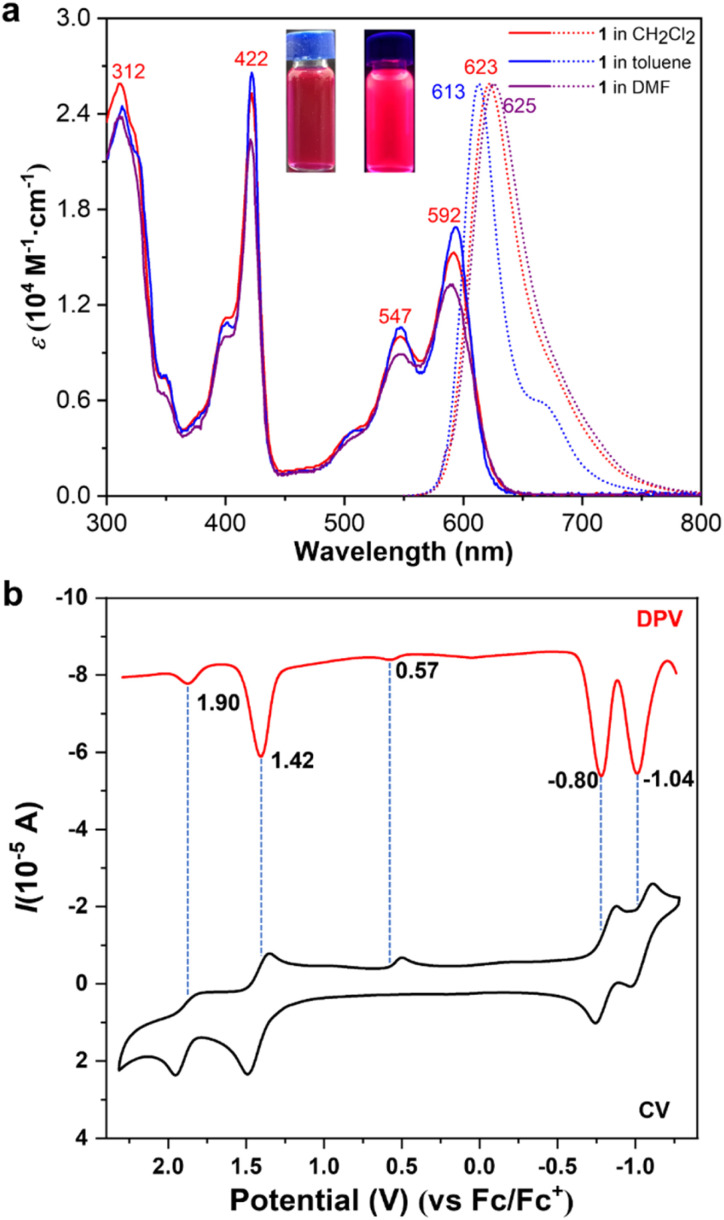
(a) UV-vis absorption (solid line, 1 × 10^−5^ M) and fluorescence emission (dotted line, 1 × 10^−5^ M, excitation at 592 nm) spectra of compound 1 in toluene, dichloromethane, and DMF. Inset: Photographs of compound 1 in dichloromethane under daylight (left) and 365 nm UV light (right). (b) Cyclic voltammetry (CV) and differential pulse voltammetry (DPV) curves of compound 1 (1 mM) in dichloromethane *versus* Fc/Fc^+^ (Fc = decamethylferrocene). Supporting electrolyte: 0.1 M ^*n*^Bu_4_NPF_6_. Working electrode: glassy carbon. Counter electrode: Pt wire. Reference electrode: Ag. Scan rate: 100 mV s^−1^.

To evaluate the optical properties of compound 1, its photophysical characteristics were systematically investigated using UV-vis absorption and fluorescence emission spectroscopy (Fig. 2). In toluene, dichloromethane, and *N*,*N*-dimethylformamide (DMF) solutions, compound 1 exhibited similar absorption bands spanning 300–650 nm, featuring four prominent peaks at 312 nm, 422 nm, and 592 nm, along with shoulder peaks at 350 nm, 400 nm, and 547 nm (Fig. 2a). Compared to bowl-shaped PAHs also containing two 6*H*-pyrrolo[3,2,1-*de*]acridin-6-one units, compound 1 demonstrated a significantly red-shifted maximum absorption wavelength.^8*c*,*f*^ This red shift is attributed to the expansion of the π-system within compound 1, suggesting a complex electronic structure with multiple electronic transitions contributing to its absorption profile. The optical energy gap, derived from the absorption onset, was estimated to be between 1.95 and 1.97 eV, corresponding to an emission wavelength range of approximately 630–635 nm, indicative of red fluorescence.

To further elucidate the observed spectral characteristics, time-dependent density functional theory (TD-DFT) calculations were performed at the B3LYP/6-311+G(d,p) level in dichloromethane. The calculated absorption spectrum closely matches the experimentally measured values (see Fig. S4†). The calculations revealed that the lowest excitation energy absorption band at 592 nm is predominantly due to the HOMO → LUMO transition, contributing 98.9% with an oscillator strength of *f* = 0.2499 (see Table S1†).

Experimentally, the HOMO–LUMO energy gap was determined through cyclic voltammetry (CV) and differential pulse voltammetry (DPV) in dichloromethane (Fig. 2b). DPV measurements yielded half-wave potentials (*E*_1/2_) of 1.90 V, 1.42 V, 0.57 V, −0.80 V, and −1.04 V, using decamethylferrocene as an external standard. These results indicate that the incorporation of multiple electron-rich heteroatoms within the bowl-shaped structure of compound 1 facilitates the stable formation of monovalent and divalent cations. Additionally, an irreversible oxidation wave was observed at *E*_1/2_ = 0.57 V. The consistency between CV and DPV redox potentials underscores the reliability of the data. The experimentally determined HOMO–LUMO energy gap of 2.22 eV aligns well with the absorption observed at 592 nm, confirming the theoretical and experimental findings.

Consistent with our predictions, compound 1 exhibits pronounced red fluorescence emission in dichloromethane, toluene, and DMF at a concentration of 1.0 × 10^−5^ M (Fig. 2a). The maximum emission peaks are observed at 613 nm, 623 nm, and 625 nm in toluene, dichloromethane, and DMF, respectively, with corresponding FWHM values of 35 nm, 50 nm, and 54 nm. Furthermore, in these solvents, the absolute fluorescence quantum yields (*Φ*_F_) were determined to be 0.57, 0.43, and 0.34, and the fluorescence lifetimes were determined to be 12.80 ns, 9.99 ns, and 9.28 ns (see Fig. S6–S8† respectively), highlighting that both the fluorescence quantum yield and the fluorescence lifetime gradually decrease with the increase in solvent polarity. Compared to bowl-shaped PAHs also containing two 6*H*-pyrrolo3,2,1-*de*acridin-6-one units, compound 1 displays a significantly red-shifted emission peak, attributable to its expanded conjugated system.^8*c*,*f*^ Furthermore, when compared to most reported nitrogen-containing buckybowls that emit red fluorescence (with maximum emission wavelengths exceeding 600 nm), including highly curved nitrogen-containing buckybowls,^7*e*^ nitrogen-containing bowl-shaped PAHs with a pyrrolopyrroles core,^8*b*^ and fully conjugated azacorannulene dimers,^12*d*^ compound 1 demonstrates a narrower FWHM and a higher fluorescence quantum yield.

Absorption and emission spectroscopy conducted at varying concentrations (1.0 × 10^−7^ M to 1.0 × 10^−5^ M) reveal no significant shifts in the positions of the absorption and emission peaks, indicating that the observed properties are solely attributed to the monomeric form of compound 1 (see Fig. S9 and S10†). The red shift in emission, the decrease in quantum yield, and the reduction in fluorescence lifetime observed in the more polar DMF solvent are attributed to intramolecular charge transfer.

**Fig. 3:**
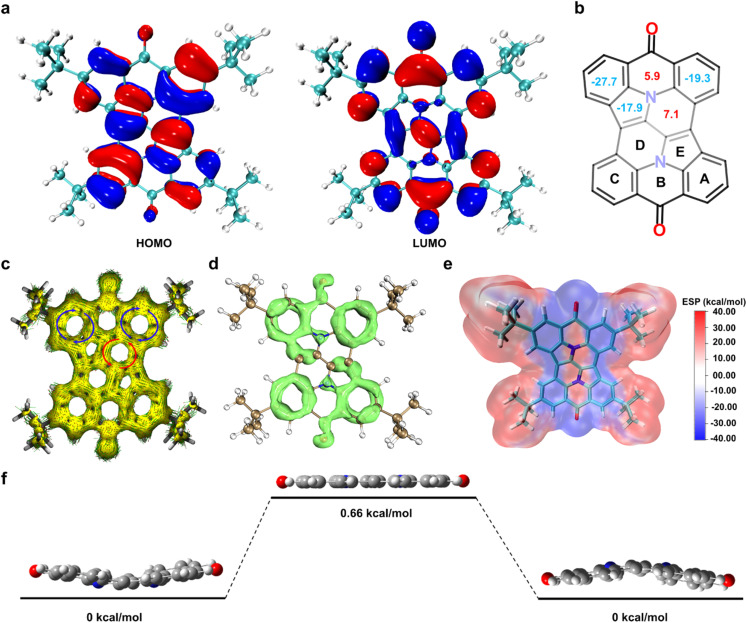
(a) DFT-calculated molecular orbitals of compound 1, viewed from the concave side, with energy levels determined using the B3LYP/6-311+G(d,p) level of theory. (b) The NICS(1)_*zz*_ values for compound 1, providing insights into its aromaticity. To clarify the structure and data, the *tert*-butyl group is omitted. (c) Calculated anisotropy of the induced current density (ACID) plots for the backbone of compound 1, illustrating delocalized electron currents. (d) Calculated localized orbital locator (LOL-π) maps of compound 1 at an isovalue of 0.5, highlighting regions of π-electron localization. (e) Electrostatic potential (ESP) map of compound 1, visualizing the charge distribution across the molecule. (f) Energy diagram depicting the inversion process of unsubstituted compound 1, showing the associated energy barriers.

To elucidate this phenomenon, density functional theory (DFT) calculations were performed at the B3LYP/6-31G(d) level in vacuum. The HOMO is delocalized across the entire molecule except for the carbonyl group, whereas the LUMO is primarily distributed over the molecule excluding the nitrogen atoms, particularly localizing on the carbonyl group (Fig. 3a). In this system, the bowl-shaped skeleton, excluding the carbonyl group, functions as the donor region, while the carbonyl unit acts as the acceptor. This distribution clarifies that the intramolecular charge transfer stems from the acceptor–donor–acceptor configuration of the molecule, which influences the optical properties of compound 1.

To assess the aromaticity of compound 1, nucleus-independent chemical shift (NICS) culations were performed using DFT at the B3LYP/6-31G(d) level (Fig. 3b).^14^ The NICS(1)_*zz*_ values indicate that the peripheral benzene rings (A and C) and the pyrrole ring (E) exhibit relatively strong aromaticity. In contrast, the six-membered rings (B and D) display weak anti-aromaticity. These findings are corroborated by anisotropy of the induced current density (ACID) analysis (Fig. 3c),^15^ which visually represents the aromatic and anti-aromatic regions within the molecule. Additionally, localized orbital locator (LOL-π) iso-surface calculations highlight electron delocalization within the aromatic benzene rings and the pyrrole ring (Fig. 3d),^16^ further supporting the NICS and ACID results. Additionally, significant π-orbital density was observed on the carbon–oxygen double bonds, reaffirming the presence of π-electron delocalization in these bonds. Electrostatic potential (ESP) analysis of 1 reveals an annular region with a relatively negative electrostatic potential within the bowl-shaped skeleton (Fig. 3e). This feature suggests that the molecule can act as a receptor for those with positively charged or less negatively charged surfaces, facilitating host–guest interactions.

Single-point energy calculations (B3LYP/6-311+G(2d,p)) on an unsubstituted model of 1 (*tert*-butyl groups replaced by hydrogen atoms for simplify the calculation) showed that the inversion proceeds *via* a planar transition state with a remarkably low energy barrier of 0.66 kcal mol^−1^ (Fig. 3f). This barrier is significantly lower than those of corannulene (10.6 kcal mol^−1^) and sumanene (19 kcal mol^−1^), highlighting the enhanced flexibility of the bowl-shaped structure in 1. The notably low inversion energy suggests that compound 1 may exhibit greater flexibility compared to other nitrogen-containing bowl-shaped molecules (see Table S2†).^6*a*,*b*,*d*,7*b*,*c*,*e*,*f*,8*b*,*d*–*f*,10,12*c*,*e*,17^

### Molecular recognition properties

Due to the extremely low inversion energy barrier of compound 1, it exhibits characteristics of both bowl-shaped and planar conformations, making it a versatile host molecule. To investigate its host–guest self-assembly properties, we selected corannulene (a bowl-shaped molecule), C_60_ (a spherical molecule), and pyrene (a planar molecule) as representative guests. Binding ratios and binding constants for each host–guest system were determined using fluorescence Job-plot analysis and titration experiments in toluene. The results reveal a consistent 1 : 1 binding ratio for all guests (see Fig. S11, S14, and S17†). Notably, the binding constants vary depending on the guest: (4.48 ± 0.17) × 10^4^ M^−1^ for corannulene (see Fig. S13†), (2.23 ± 0.01) × 10^3^ M^−1^ for C_60_ (see Fig. S16†), and (3.44 ± 0.11) × 10^4^ M^−1^ for pyrene (see Fig. S19†). These findings suggest that compound 1 demonstrates a preferential binding affinity for guests with bowl-shaped and planar geometries, likely due to complementary conformational interactions.

The results demonstrate that nitrogen-containing buckybowl 1 uniquely exhibits the ability to bind both concave and planar molecules, a property unprecedented among previously studied bowl-shaped molecules. This dual binding capability underscores the versatility of compound 1 in host–guest chemistry. It is noteworthy that the relatively low degree of curvature in 1 reduces its compatibility with C_60_, resulting in a binding constant significantly lower than that observed with corannulene or pyrene. However, its binding constant with C_60_, measured at 2.23 × 10^3^ M^−1^, is lower than those of other nitrogen-containing bowl-shaped PAHs (*e.g.*, 3800 M^−1^, 44 300 M^−1^),^7*b*,12*e*^ yet despite its geometric constraints, it still highlights its competitive binding efficiency.

During the fluorescence titration experiments, the fluorescence intensity of compound 1 was found to increase progressively with the addition of corannulene or pyrene (see Fig. S12 and S18†), whereas the fluorescence intensity gradually decreased with increasing concentrations of C_60_ (see Fig. S15†). This fluorescence enhancement is attributed to the restriction of the host molecule's degrees of freedom upon complexation with rigid, curved, or planar guest molecules. This restriction likely reduces non-radiative vibrational relaxation processes, resulting in greater energy release as fluorescence. Conversely, the observed fluorescence quenching with C_60_ is consistent with the behavior of other nitrogen-containing bowl-shaped PAHs and is primarily attributed to intermolecular charge transfer between compound 1 and C_60_,^7*b*^ which facilitates non-radiative energy dissipation.

**Fig. 4:**
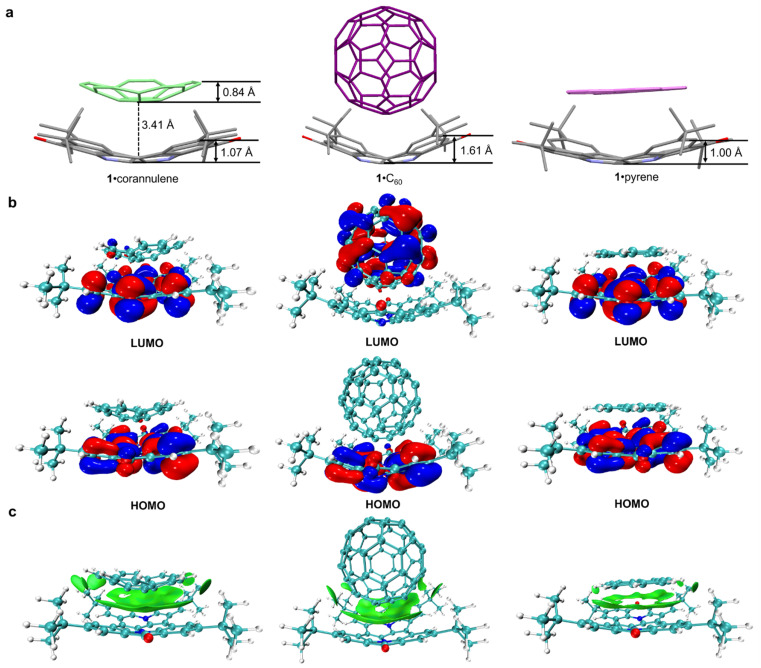
(a) Optimized structure of the host–guest complex, with the bowl depth of compound 1 highlighted. (b) DFT-calculated molecular orbitals of compound 1, viewed from the side. (c) Independent Gradient Model based on Hirshfeld (IGMH) analysis, illustrating interaction regions. The bowl depth of compound 1 is defined as the distance from the carbon center at the junction of the four *tert*-butyl groups to the center of the C–C single bond at the bottom of the bowl. In (a), corannulene, C_60_, and pyrene are depicted in green, purple, and pink, respectively. Hydrogen atoms are omitted for clarity.

To further elucidate the interactions between compound 1 and its guests, density functional theory (DFT) calculations incorporating Grimme's D3 dispersion correction were performed at the B3LYP/6-31G(d) level to study the complexation. The optimized structures reveal that corannulene, C_60_, and pyrene are all centrally positioned on the concave surface of compound 1 (see Fig. S20†), with corresponding bowl depths of 1.07 Å, 1.61 Å, and 1.00 Å, respectively (Fig. 4a). Notably, the curvature of compound 1 adapts to the structural characteristics of each guest, demonstrating its flexible assembly performance.

In terms of electronic structure, the HOMO and LUMO of the 1·corannulene and 1·pyrene complexes are delocalized entirely within molecule 1, with no contribution from the guest molecules (Fig. 4b). In contrast, in the 1·C_60_ complex, the HOMO is delocalized throughout molecule 1, while the LUMO is predominantly localized on C_60_. This differential electronic distribution aligns with the fluorescence titration results, where fluorescence quenching in the presence of C_60_ can be attributed to charge transfer between the host and guest, whereas fluorescence enhancement with corannulene and pyrene results from restricted vibrational relaxation.

Additionally, IGMH analysis identified bright green regions (Fig. 4e), indicative of weak interactions predominantly governed by π–π stacking between host 1 and all guests. Among them, the π–π stacking surfaces between 1 and corannulene or C_60_ are distributed on the core concave surface. However, in the case of pyrene, they are only distributed on the peripheral ring part surrounding the core of the bowl. These computational findings provide a deeper understanding of the host–guest interactions, highlighting the significance of π–π donor–acceptor interactions in stabilizing the host–guest complex. ESP analysis provided additional insight, showing a relatively negative annular region in the bowl-shaped skeleton of 1 and a corresponding positive region in guests (see Fig. S21†). This complementary electrostatic potential distribution highlights the role of electrostatic attraction in stabilizing the host–guest complex, enhanced by the electron-rich heteroatoms in 1. Relative distribution gradient (RDG) analysis, performed using Multiwfn software,^18^ confirmed the presence of strong non-covalent forces, consistent with experimental observations (see Fig. S22†). Such insights underscore the potential of compound 1 in applications requiring precise molecular assemblies, particularly within the realms of nanotechnology and materials science.

**Fig. 5:**
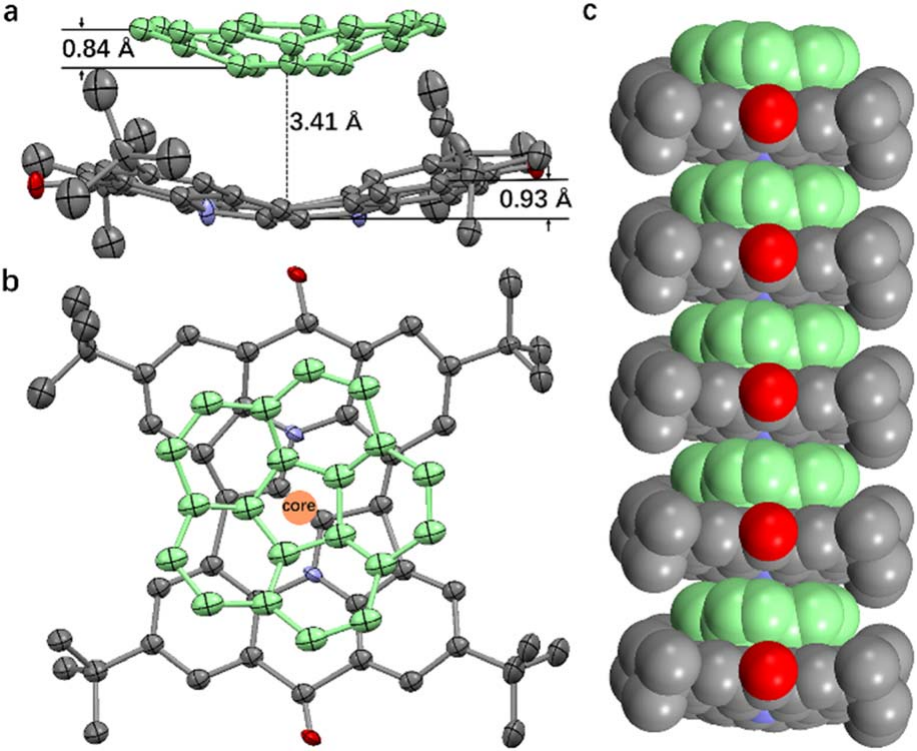
(a) ORTEP diagram of the 1·corannulene complex, with thermal ellipsoids shown at the 30% probability level. (b) Overlay of the core structures of compound 1 and corannulene within the single-crystal structure of 1·corannulene. (c) One-dimensional self-assembled structure formed by compound 1 and corannulene. Corannulene is depicted in green, and hydrogen atoms are omitted for clarity.

Further evidence of host–guest interactions was obtained through single-crystal X-ray diffraction analysis. Light green cubic crystals of the 1·corannulene complex were prepared by slow evaporation of a dichloromethane/hexane solution containing 1 and corannulene. Structural analysis confirmed the formation of a 1 : 1 complex between 1 and corannulene (see Fig. 5a and S23†). In this complex, the bowl depths of compound 1 and corannulene were measured at 0.93 Å and 0.84 Å, respectively. The bowl depth of compound 1 is slightly lower than the theoretical value (1.07 Å), whereas that of corannulene aligns with the theoretical value (0.84 Å). Consistent with computational predictions (see Fig. S20†), the concave surfaces of compound 1 and corannulene are aligned in the same direction, with their cores positioned along a straight axis (Fig. 5b). The measured face-to-face distance between the bottoms of the two bowls is 3.41 Å, indicative of strong π–π donor–acceptor interactions between the host and guest molecules. The agreement between single-crystal data and theoretical calculations reinforces the validity of the proposed adaptive self-assembly mechanism.

Additionally, the crystal structure reveals that compound 1 and corannulene assemble into a one-dimensional array *via* bowl-to-bowl stacking (see Fig. 5c and S24†). This highly ordered arrangement underscores the potential of compound 1·curved molecule complexes for applications in nanotechnology and materials science, where precise molecular organization is essential for functional material design.

## Conclusions

In conclusion, we successfully synthesized nitrogen-containing buckybowls with a C–C single bond at the core, employing a core-periphery strategy to link two planar precursors *via* single bonds. This compound demonstrates solvent-dependent red-light emission with high color purity and quantum yield, showcasing its potential for optoelectronic applications. Moreover, the electron-rich core and extremely low flipping energy barrier enable the buckybowl 1 to self-assemble with bowl-shaped corannulene, spherical C_60_, and planar pyrene through strong non-covalent interactions, including π–π donor–acceptor interactions, in solution. These experimental observations are corroborated by theoretical calculations, which confirm the geometric and electronic compatibility of the host–guest interactions. This study underscores the potential of the single-bond core strategy for constructing bowl-shaped molecules, presenting a novel approach to their design and synthesis. The compound's excellent red light emission performance offers new avenues for developing organic molecules for functional optical materials. Additionally, the observed one-dimensional columnar assembly with corannulene provides fresh insights into advanced assembly strategies for bowl-shaped molecules. Collectively, these findings not only expand the molecular design toolkit but also lay the foundation for exploring functional materials based on bowl-shaped architectures and their supramolecular assemblies.

## Data availability

Data supporting the findings of this study are available within the main text and its ESI† or from the corresponding author upon request. The crystallographic data corresponding to the 1·corannulene complex have been submitted to the Cambridge Crystallographic Data Centre (CCDC) and assigned the deposition number 2412345. These data can be retrieved by accessing the following URL: https://www.ccdc.cam.ac.uk/solutions/csd-core/components/csd/.

## Author contributions

X.-L. C. and S.-Q. Y. conducted the experimental work and prepared the initial manuscript under the guidance of H.-Y. G. and X.-H. H. Z.-Y. Z., Z.-Y. C., and A.-N. C. provided valuable assistance with the synthesis of compounds. J.-Q. L. and X. S. contributed to experimental testing and analysis, particularly in the optical studies. C.-Y. Z. supported data collection and analysis for the theoretical calculations. The project was designed by X.-L. C. and H.-Y. G., who also contributed to manuscript preparation. H.-Y. G. and X.-L. C. were responsible for the initial discoveries and supervised the crystallographic studies.

## Conflicts of interest

There are no conflicts to declare.

## Supplementary Material

SC-OLF-D5SC00988J-s001

SC-OLF-D5SC00988J-s002
