# Supplementary material for: Adaptive nitrogen-containing buckybowl: a versatile receptor for curved and planar aromatic molecules
Source: Chem Sci. 2025 Mar 25;16(17):7537–43. doi: 10.1039/d5sc00988j (PMC11955802; doi:10.1039/d5sc00988j)
Supplement: SC-016-D5SC00988J-s001 [file SC-016-D5SC00988J-s001.pdf]

Supplementary Information (SI) for Chemical Science.
This journal is © The Royal Society of Chemistry 2025

Supplementary Information For:

Adaptive Nitrogen-Containing Buckybowl: A Versatile Receptor for Curved and Planar Aromatic Molecules

Xu-Lang Chen,*[a] Si-Qian Yu,[a] Zi-You Zheng,[a] Zhao-Yi Cheng,[a] An-Na Chen,[a] Jia-Qi Liang,[b] Xin Sun,[c] Chunyang Zheng,*[a] Xiaohuan Huang,*[a] and Han-Yuan Gong*[b]

a College of Chemistry and Chemical Engineering, Hubei Key Laboratory of Pollutant Analysis and Reuse Technology, Hubei Normal University, Huangshi, 435002, P. R. China; E-mail: xulangchen@hbnu.edu.cn; cyzheng@hbnu.edu.cn; xhuang@hbnu.edu.cn.

b College of Chemistry, Beijing Normal University, No. 19, Xin Jie Kou Wai St, Hai Dian District, Beijing, 100875, P. R. China; E-mail: hanyuangong@bnu.edu.cn.

c College of Materials Chemistry & Chemical Engineering, Chengdu University of Technology, 1#, Dongsanlu, Erxianqiao, Chengdu, 610059, P. R. China.

Table of Content

1. General remarks……………………………………………………..….S3

2. The synthesis of compound 1………………………………………………S4

3. NMR spectra ……………………………………………………………….S5

4. Mass spectra ………………………………………………………………..S6

5. Theoretical calculations on 1………………………………………………S7

6. Electrochemical Study……………………………………………………...S9

7. The fluorescence lifetime of 1……………………………………………..S10

8. Uv-vis absorption and fluorescence spectra of 1 at different concentrations…………………………………………………………..S11

9. Summary of inversion energy barriers…………………………………..S12

10. Association behavior of 1 with corannulene……………………………S14

11. Association behavior of 1 with C_{60}……………………………………....S15

12. Association behavior of 1 with pyrene…………………………………...S17

13. Theoretical calculations on the host-guest complex……………………S18

14. X-ray crystallographic structure determination………………………S22

15. Cartesian coordinates for theoretically optimized structures…………S24

16. References………………………………………………………….…..S40

1. General remarks

All the reagents and solvents used in this study were purchased from Energy Chemical, Leyan, and Tianjin Damao Chemical Reagent Factory. Among them, the extra-dry DMA and CH_2Cl_2 was purchased from Energy Chemical (99.8% and 99.9% Extra Dry, Water ≤ 50 ppm (by K.F.), EnergySeal) and the $Pd(PCy_3)_2Cl_2$ (98%, Cat No. 1151409, Leyan, Shanghai, China) was purchased from Leyan. Other chemicals can be used without further purification. The compound 2,2′-dibromo-4,4′,8,8′-tetra-*tert*-butyl-[1,1′-bipyrrolo[3,2,1-*de*]acridone-6,6′-dione (**2**) was synthesized following a previously reported method.[S1] Flash column chromatography was performed using silica gel (particle size: 200–300 mesh).

Characterization of the synthesized compounds involved various analytical techniques. Melting points were determined using an X-4 Digital Display Micro Melting Point Tester. The ^{1}H NMR and ^{13}C NMR spectra were recorded on a Bruker Avance 300 MHz spectrophotometer using $CDCl_3$ as a solvent. Chemical shifts (δ) reported in parts per million (ppm) relative to residual $CHCl_3$ in deuterated solvents. The terms s represent singlet. High-resolution mass spectrometry (HRMS) was conducted using a Bruker Autoflex Speed MALDI-TOF instrument. Uv-vis absorption spectra were obtained using a HITACHI U-3900 spectrophotometer, and fluorescence emission spectra were recorded on a HITACHI F-4600 fluorescence spectrophotometer. Time-resolved fluorescence spectra were measured by time-correlated single-photon counting using an Edinburgh Instruments FLS-980 spectrometer. Fluorescence quantum yields were determined with a HAMAMATSU C11347 quantum yield measurement system.

The binding constants of the host-guest systems were calculated using an online tool available at http://supramolecular.org, following previously established methodologies.[S2,S3]

Single-crystal X-ray diffraction data for the **1**•corannulene complex were collected on an XtaLAB Synergy R, HyPix. Data reduction was performed using CrysAlisPro 1.171.41.121a (Rigaku OD, 2021). The structures were solved via direct methods and refined using full-matrix least-squares on F^2 with anisotropic displacement parameters for non-hydrogen atoms, employing the SHELXL2018 software.[S4] The R(F), $R_w(F^2)$, and goodness-of-fit (S) values are provided in the accompanying .cif file. The .cif file also contains detailed tables of positional and thermal parameters, bond lengths and angles, torsion angles, structure factors, and additional crystallographic data, including details of the data collection and structure refinement. This file is available from the Cambridge Crystallographic Data Centre (CCDC) by quoting deposition number 2412345.

Cyclic voltammetry (CV) and differential pulse voltammetry (DPV) measurements were conducted using a glassy carbon working electrode, a platinum counter electrode,

and a silver wire reference electrode on a CHI660E electrochemical workstation. Electrochemical data were obtained in an anaerobic extra-dry CH_2Cl_2 solution containing 0.1 M tetrabutylammonium hexafluorophosphate, with decamethylferrocene serving as an internal standard.

2. The synthesis of compound 1

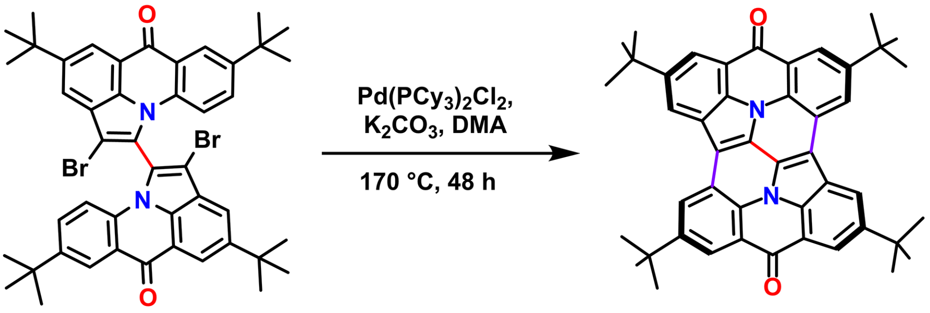

A 350 mL glass reaction tube was charged with compound **2** (5 g, 6.1 mmol), $Pd(PCy_3)_2Cl_2$ (dichlorobis(tricyclohexylphosphine)palladium(II), 902 mg, 1.22 mmol), K_2CO_3 (5.06 g, 36.6 mmol). Under a nitrogen atmosphere, dry *N*,*N*-dimethylacetamide (DMA, 120 mL) was added to the reaction mixture. The tube was then heated to 170 °C and maintained at this temperature for 48 hours. After cooling to room temperature, the reaction mixture was concentrated by rotary evaporation and quenched with water. The crude mixture was diluted with dichloromethane (200 mL), washed with water (200 mL × 3), and dried over anhydrous sodium sulfate (Na_2SO_4). Following solvent removal via rotary evaporation, the crude product was purified by silica gel column chromatography (200-300 mesh) using a dichloromethane/petroleum ether (1:1, *v*/*v*) solvent system, yielding compound **1** (160 mg, 4%) as purple solids.

Compound **1**: m.p. > 300°C. ^{1}H NMR (300 MHz, $CDCl_3$) δ (ppm) = 8.31 (s, 2H), 8.24 (s, 2H), 8.21 (s, 2H), 8.05 (s, 2H), 1.56 (s, 18H), 1.54 (s, 18H). ^{13}C NMR (75 MHz, $CDCl_3$) δ (ppm) = 179.7, 148.7, 148.1, 133.5, 130.2, 130.1, 126.4, 125.6, 124.9, 124.2, 123.7, 121.9, 120.2, 119.8, 104.2, 35.9, 35.28, 32.3, 31.7. HRMS (MALDI-TOF) (*m*/*z*): $[M]^+$ calcd. for $C_{46}H_{44}N_2O_2$, 656.3397; found, 656.3398

3. NMR spectra

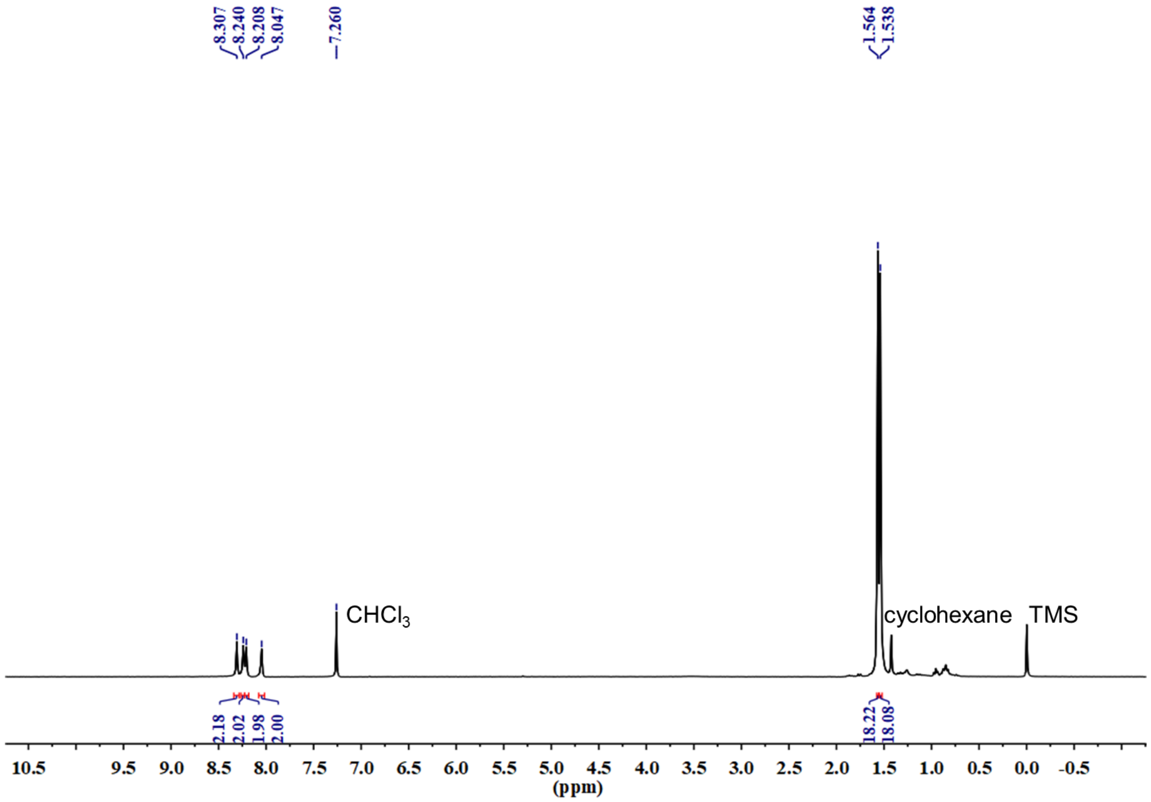

Fig. S1. ^{1}H NMR spectrumof **1** (1.0×10^{-2} M, $CDCl_3$, 298K, 300 MHz).

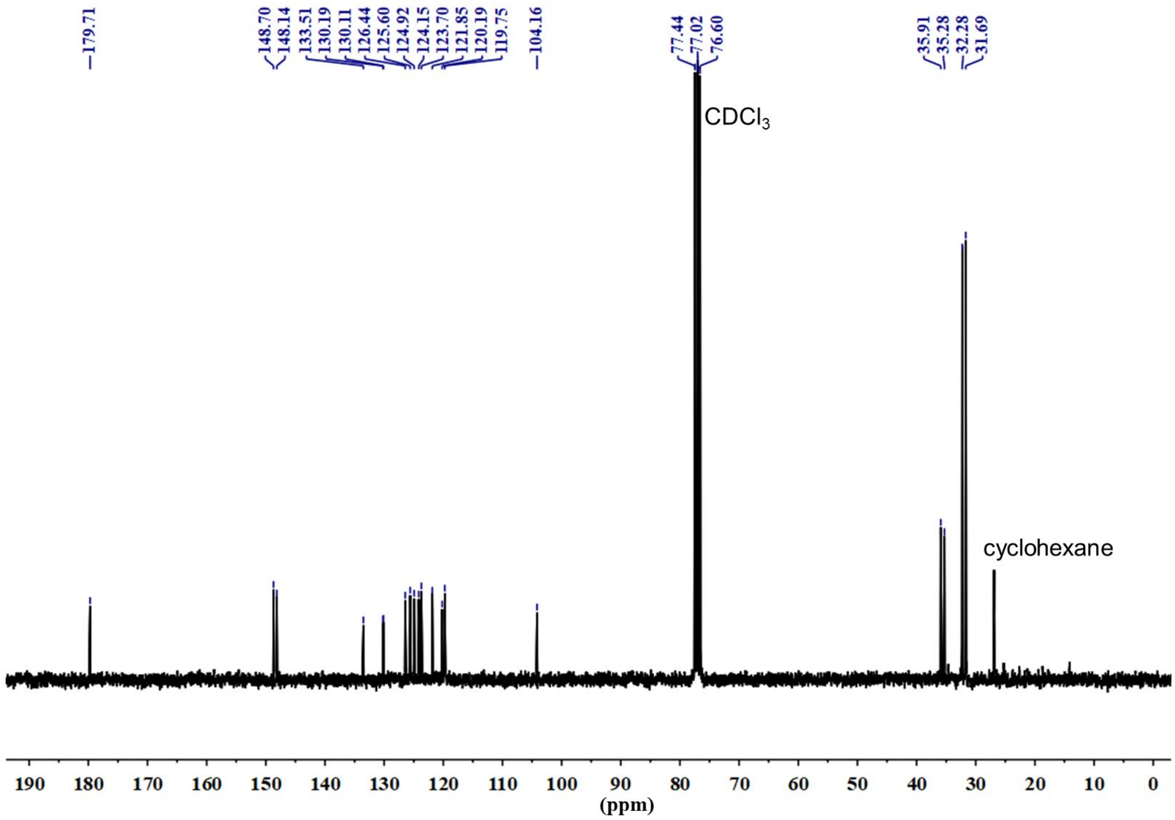

Fig. S2. ^{13}C NMR spectrumof **1** (1.0×10^{-2} M, $CDCl_3$, 298K, 75 MHz).

4. Mass spectra

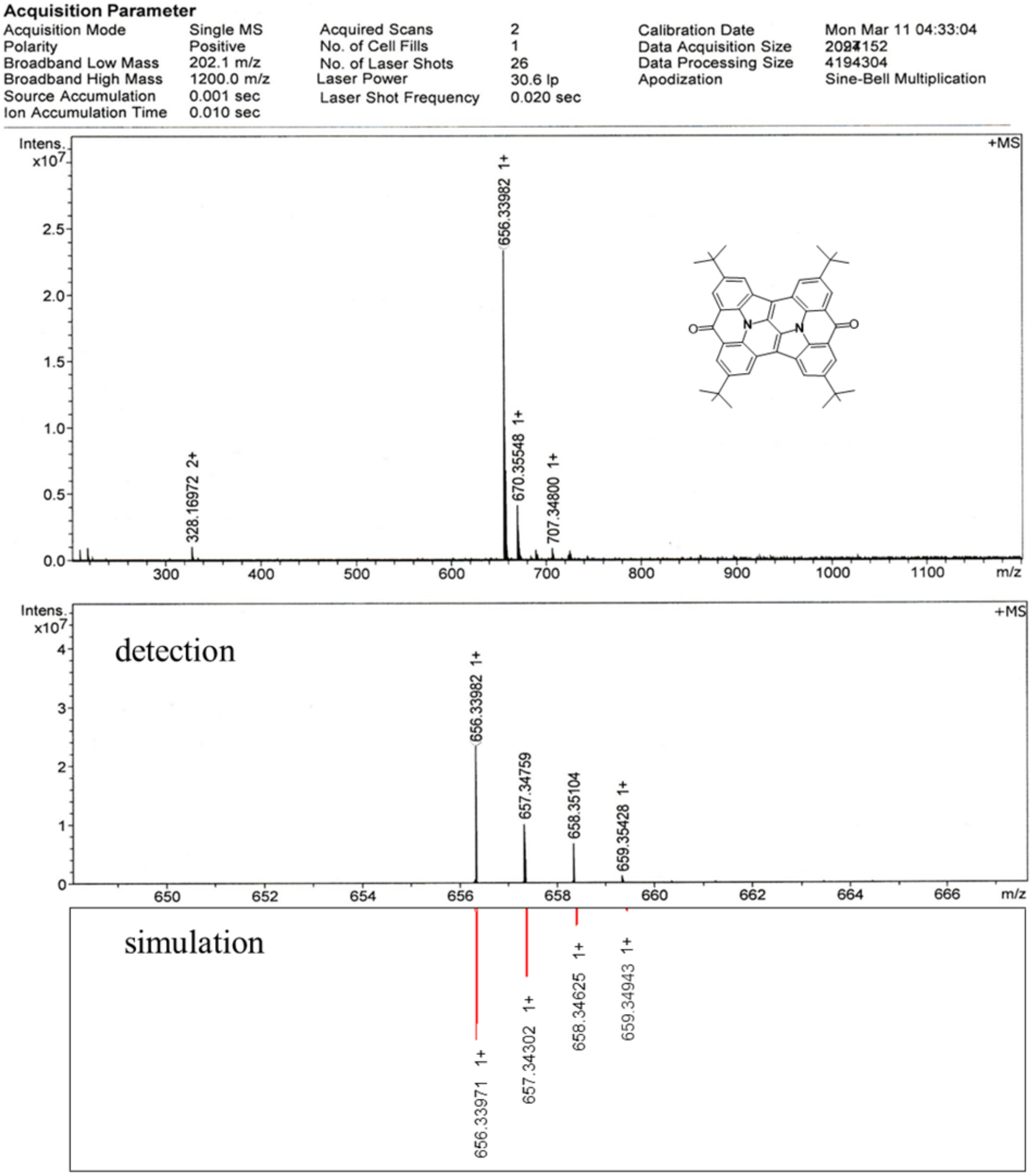

Fig. S3. Positive HRMS spectrum of **1**. HRMS (MALDI-TOF) (*m*/*z*): $[M]^+$ calcd. for $C_{46}H_{44}N_2O_2$, 656.3397; found, 656.3398.

5. Theoretical calculations on 1

All theoretical calculations were performed using Gaussian 16 software.[S5] Geometry optimizations and frontier molecular orbital analyses were conducted at the B3LYP/6-31G(d) level of theory. Bowl-to-bowl inversion energies were calculated using single-point energy evaluations at the B3LYP/6-311+G(2d,p) level, with planar transition states verified through frequency calculations at the B3LYP/6-31G(d) level.[S6] Excited-state properties were analyzed using time-dependent density functional theory (TD-DFT) at the B3LYP/6-311+G(d,p) level, incorporating solvent effects with CH_2Cl_2 as the medium.

Nucleus-independent chemical shift (NICS) calculations were performed at the GIAO-B3LYP/6-31G(d) level of theory. Bq atoms were positioned 1 Å above the molecule for these calculations, with their coordinates fixed using Multiwfn 3.8 software.[S7] Localized orbital locator (LOL-π) isosurfaces, independent gradient model based on Hirshfeld partitioning (IGMH), and reduced density gradient (RDG) analyses were also conducted using Multiwfn, following established protocols from the literature.[S7a,S8]

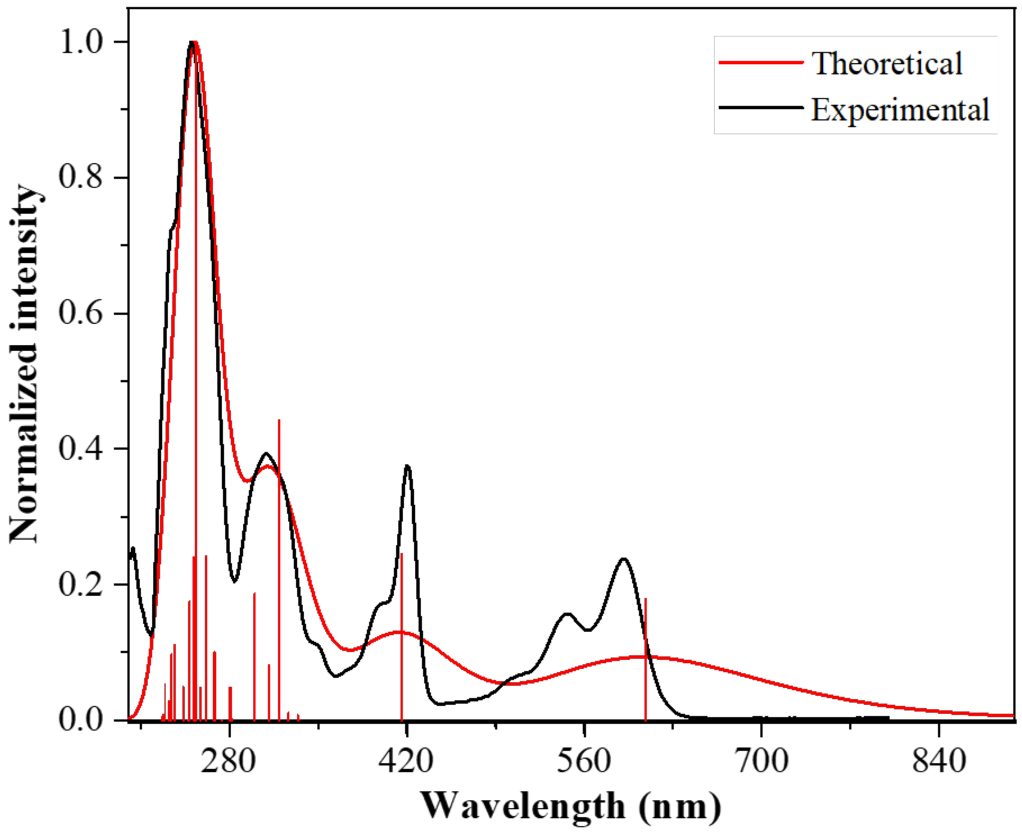

Fig. S4. Simulated absorption spectrum of compound **1** (red solid line), calculated at the B3LYP/6-311+G(d,p) level of theory, compared with the experimental absorption spectrum (black solid line).

Table S1. TD-DFT-calculated first ten electronic transitions of compound **1** in CH_2Cl_2, computed at the B3LYP/6-311+G(d,p) level of theory.

Excited State	1:	Singlet-A	2.0383 eV	608.29 nm	f=0.2499
<S**2>=0.000					
175 -> 176		98.9%			
Excited State	2:	Singlet-A	2.4279 eV	510.66 nm	f=0.0000
<S**2>=0.000					
175 -> 177		99.0%			
Excited State	3:	Singlet-A	2.9814 eV	415.85 nm	f=0.3421
<S**2>=0.000					
174 -> 176		96.0%			
Excited State	4:	Singlet-A	3.2633 eV	379.93 nm	f=0.0004
<S**2>=0.000					
173 -> 176		47.2%			
175 -> 178		49.3%			
Excited State	5:	Singlet-A	3.3747 eV	367.40 nm	f=0.0002
<S**2>=0.000					
173 -> 176		51.2%			
174 -> 177		3.0%			
175 -> 178		44.1%			
Excited State	6:	Singlet-A	3.4841 eV	355.86 nm	f=0.0020
<S**2>=0.000					
174 -> 177		91.8%			
175 -> 178		4.7%			
Excited State	7:	Singlet-A	3.5733 eV	346.97 nm	f=0.0000
<S**2>=0.000					
167 -> 176		2.7%			
168 -> 177		22.3%			
169 -> 176		62.3%			
170 -> 177		3.9%			
171 -> 176		2.2%			
172 -> 176		2.7%			
Excited State	8:	Singlet-A	3.5950 eV	344.88 nm	f=0.0010
<S**2>=0.000					
168 -> 176		53.8%			
169 -> 177		27.9%			
170 -> 176		11.7%			

Excited State 9: Singlet-A 3.7140 eV 333.83 nm f=0.0168
<S**2>=0.000
173 -> 177 8.6%
175 -> 179 87.9%
Excited State 10: Singlet-A 3.7961 eV 326.61 nm f=0.0214
<S**2>=0.000
173 -> 177 76.2%
175 -> 179 6.2%
175 -> 180 12.9%

HOMO:175 LUMO:176

6. Electrochemical Study

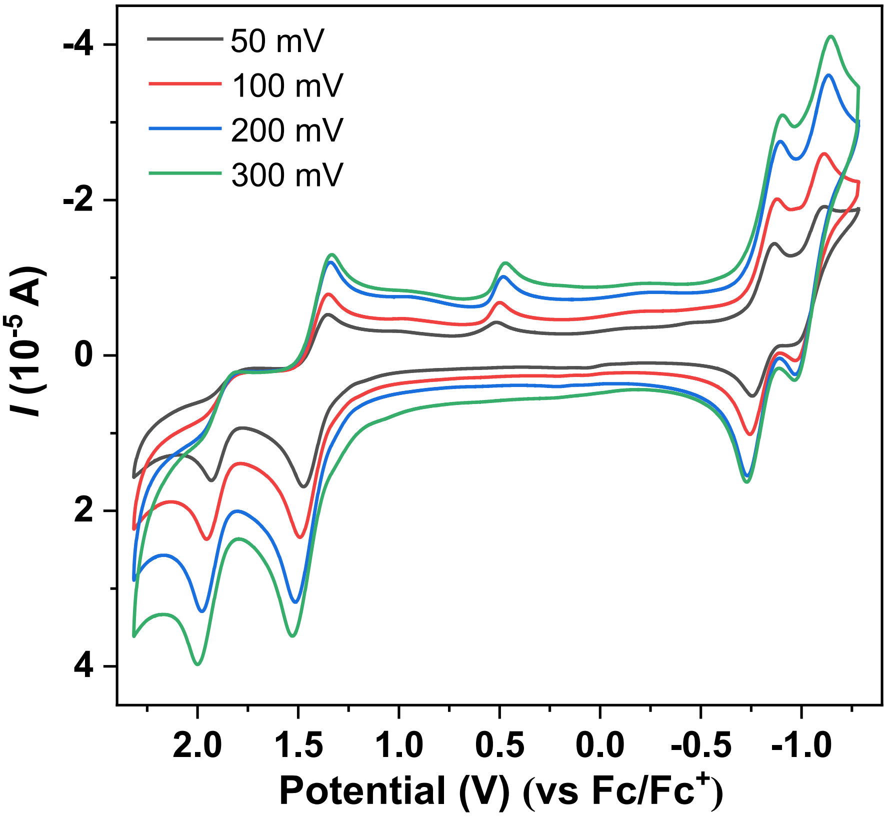

Fig. S5. Cyclic voltammetry (CV) curves of compound **1** (1.0 × 10^3 M, *vs* Fc/Fc^+; Fc = decamethylferrocene) in CH_2Cl_2 at varying scan rates, illustrating the quasi-reversibility of the redox processes.

7. The fluorescence lifetime of 1

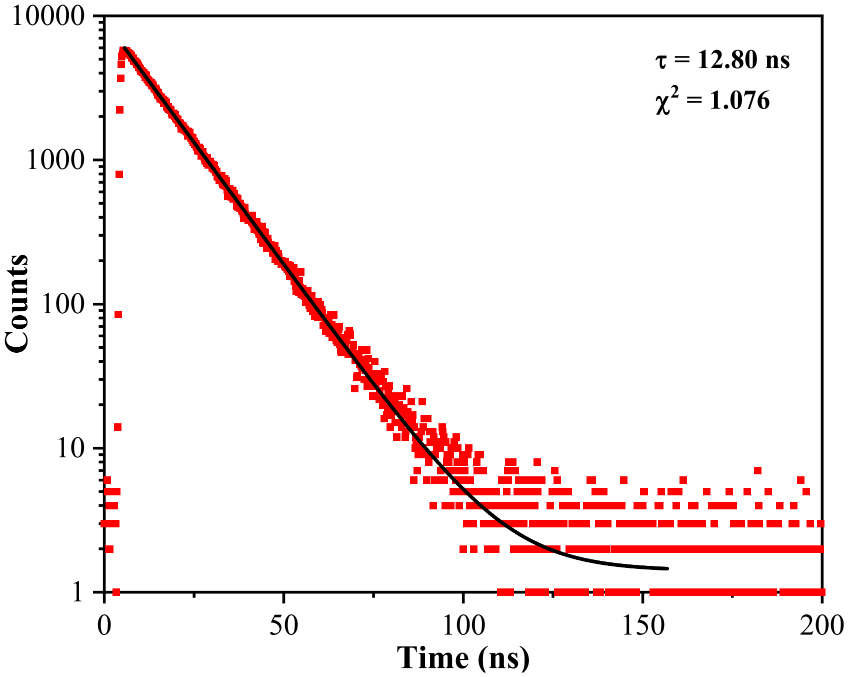

Fig. S6. Fluorescence decay plot of compound **1** in toluene at room temperature ([**1**] = 1.0 × 10^{-5} M), with decay parameters τ_1 = 12.80 ns, A_1 = 100%, x^2 = 1.076.

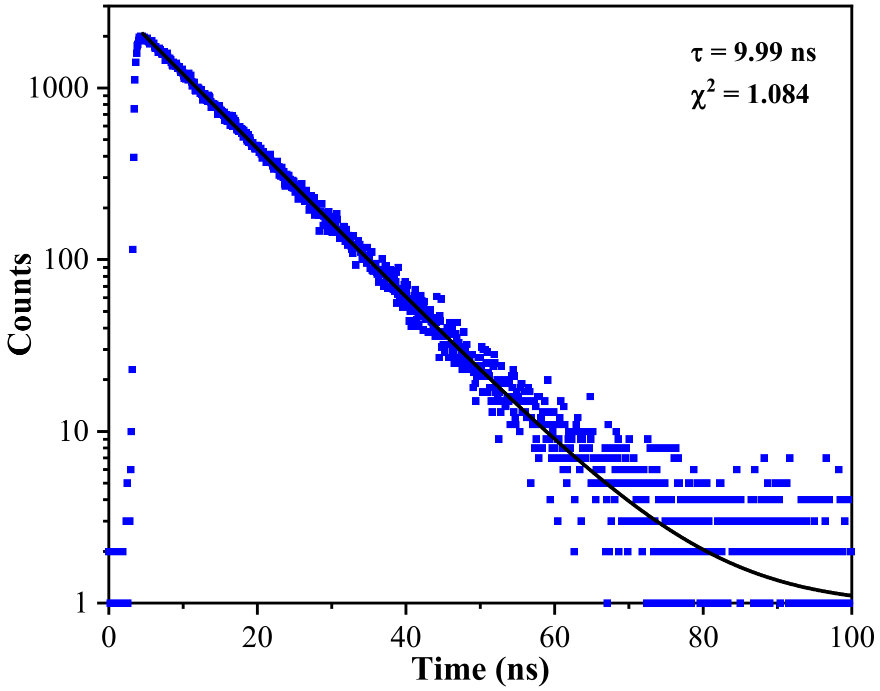

Fig. S7. Fluorescence decay plot of compound **1** in CH_2Cl_2 at room temperature ([**1**] = 1.0 × 10^{-5} M), with decay parameters τ_1 = 9.99 ns, A_1 = 100%, x^2 = 1.084.

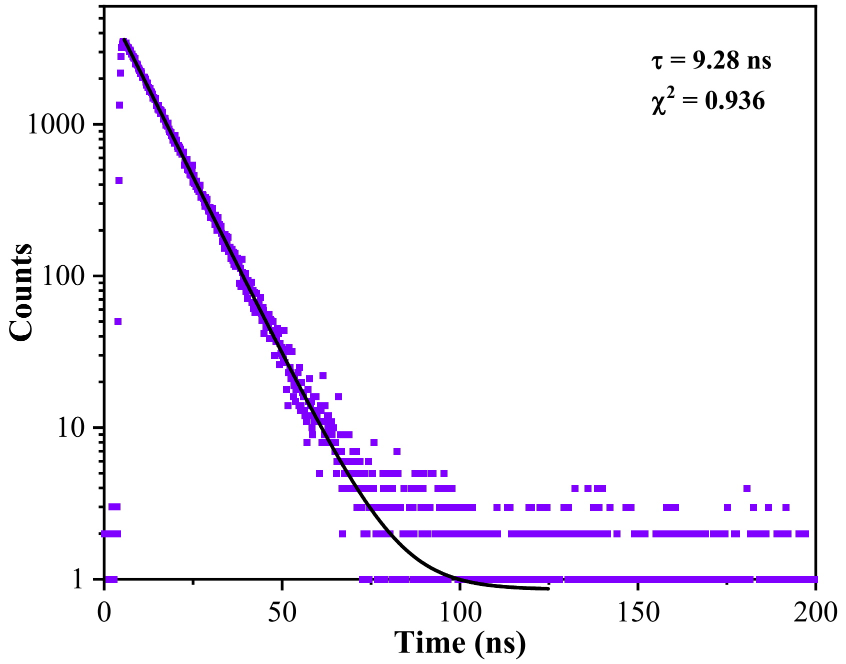

Fig. S8. Fluorescence decay plot of compound **1** in DMF at room temperature ([**1**] = 1.0 × 10^{-5} M), with decay parameters τ_1 = 9.28 ns, A_1 = 100%, x^2 = 0.936.

8. Uv-vis Absorption and Fluorescence spectra of 1 at different concentrations

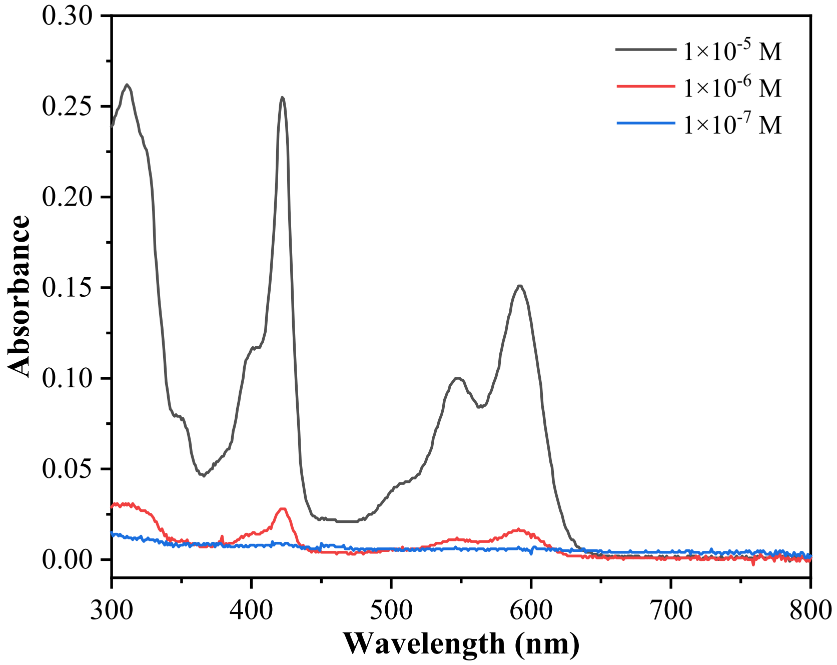

Fig. S9. Uv-vis absorption spectra of compound **1** in CH_2Cl_2 at varying concentrations ([**1**] = 1 × 10^{-7} M, 1 × 10^{-6} M, 1 × 10^{-5} M).

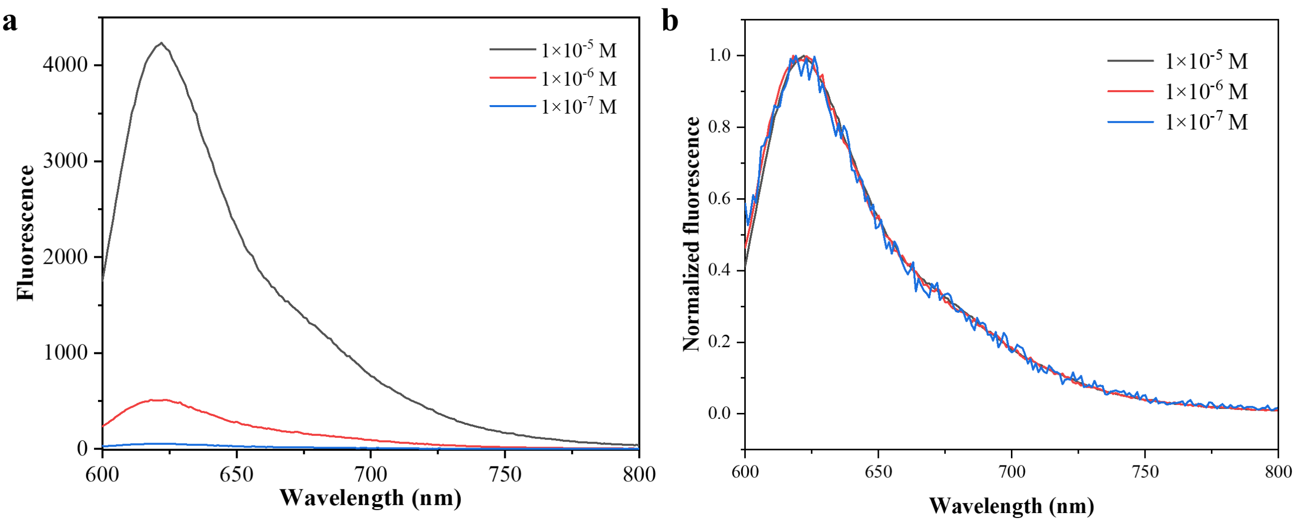

Fig. S10. Fluorescence emission spectra of compound **1** in CH_2Cl_2 at varying concentrations ([**1**] = 1.0 × 10^{-7} M, 1.0 × 10^{-6} M, 1.0 × 10^{-5} M; a) and their normalized results (b).

9. Summary of inversion energy barriers

Table S2. Summary of inversion energy barriers for published nitrogen-containing bowl-shaped PAHs.

The skeletal structure of nitrogen-containing bowl-shaped PAHs[a]	Energy barrier (kcal mol^{-1})	The skeletal structure of nitrogen-containing bowl-shaped PAHs[a]	Energy barrier (kcal mol^{-1})
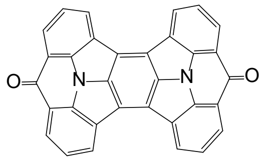 ref. 6a	11.2	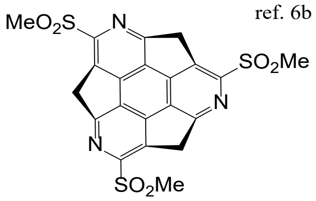 ref. 6b	42.2
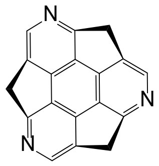 ref. 6d	37.9	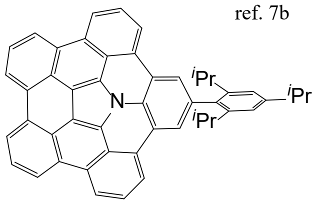 ref. 7b	19.9
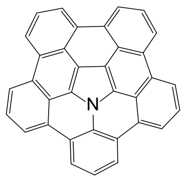 ref. 7c	15.3-17.0	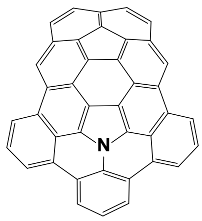 ref. 7e	79.0

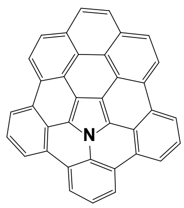 ref. 7f	23.7	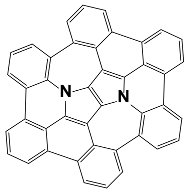 ref. 8b	5.4
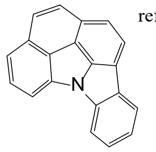 ref. 8d	2.1	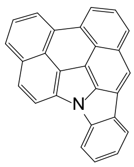 ref. 8d	4.5
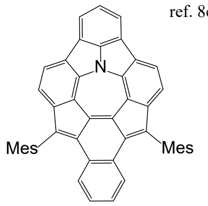 ref. 8e	7.5	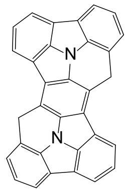 ref. 8f	20.2
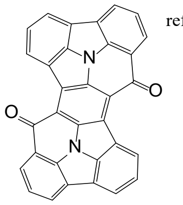 ref. 8f	14.5	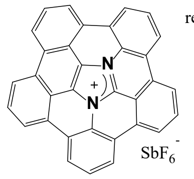 ref. 10	8.5
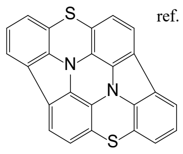 ref. 12c	3.6	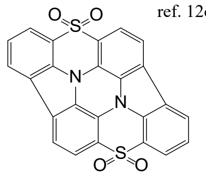 ref. 12c	3.8
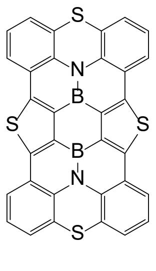 ref. 12e	8.6	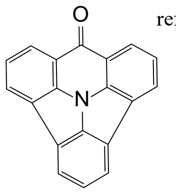 ref. 17a	8.1
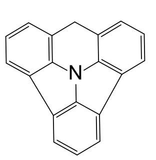 ref. 17a	11.6	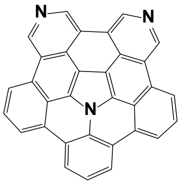 ref. 17b	19.9
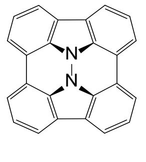 ref. 17c	2.0	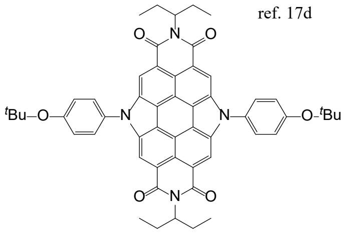 ref. 17d	15.22

[a]The literature numbers in the upper right corner correspond to the literature citation numbers in the main text.

10. Association behavior of 1 with corannulene

In this study, "H" (Host) refers to compound **1**, while "G" (Guest) denotes corannulene. These abbreviations will be used throughout the text for clarity and consistency.

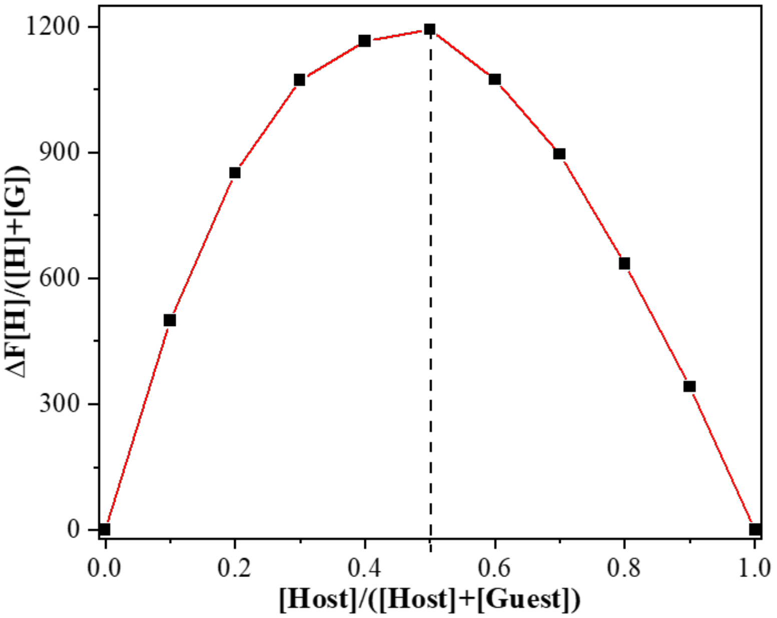

Fig. S11. Job plot for the complexation of compound **1** and corannulene, derived from fluorescence titration experiments in toluene ([H] + [G] = 1×10^{-5} M). The maximum value was found at 0.5, a finding consistent with (but not a proof of) a 1:1 (host:guest) binding stoichiometry.[S9]

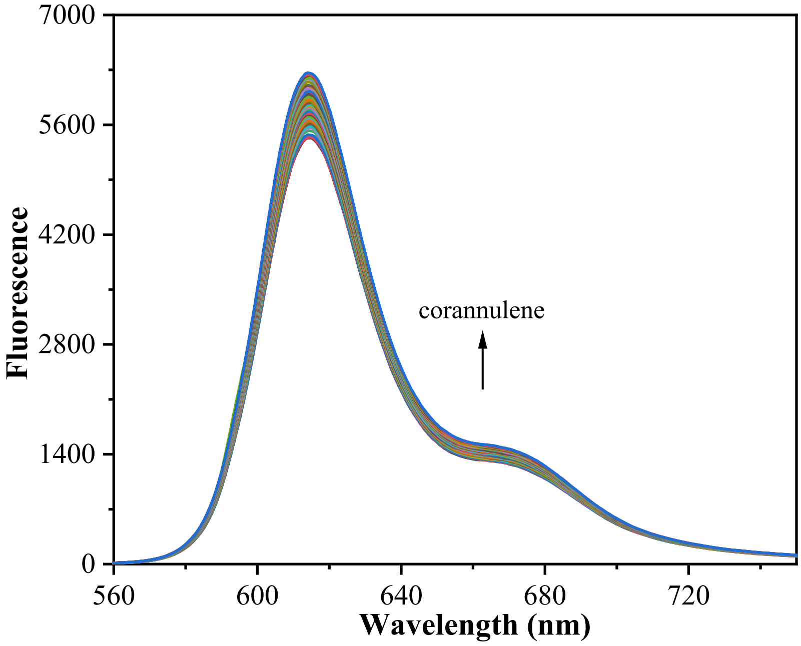

Fig. S12. Fluorescence emission spectra of compound **1** ([**1**] = 1×10^{-5} M) in toluene upon the incremental addition of corannulene (0 - 20 molar equiv.).

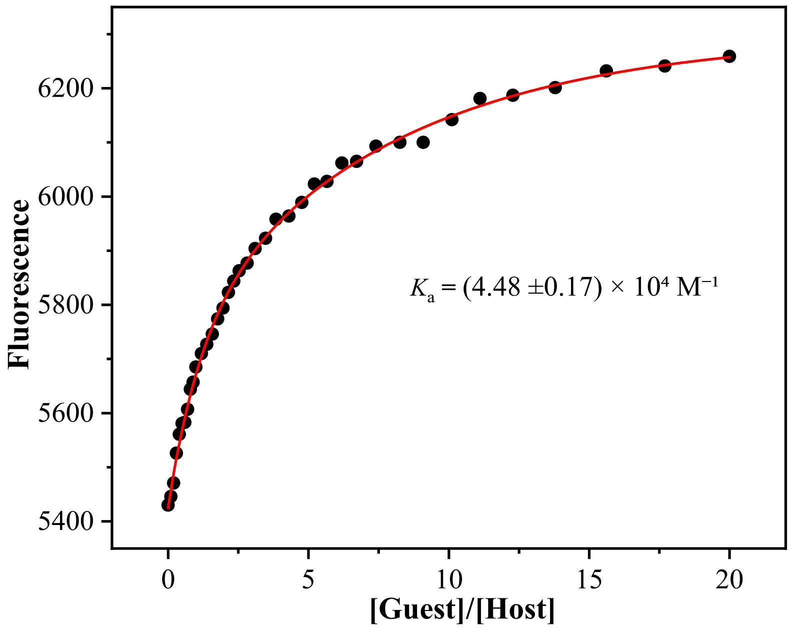

Fig. S13. Binding constant of compound **1** and corannulene, determined from fluorescence titration measurements in toluene (λ_{em} = 614 nm). Calculation results are accessible at http://app.supramolecular.org/bindfit/view/3ce8b424-d7d3-4efb-b44b-40375bb69277.

11. Association behavior of 1 with C_{60}

In this study, "H" (Host) refers to compound **1**, while "G" (Guest) denotes C_{60}. These abbreviations will be used throughout the text for clarity and consistency.

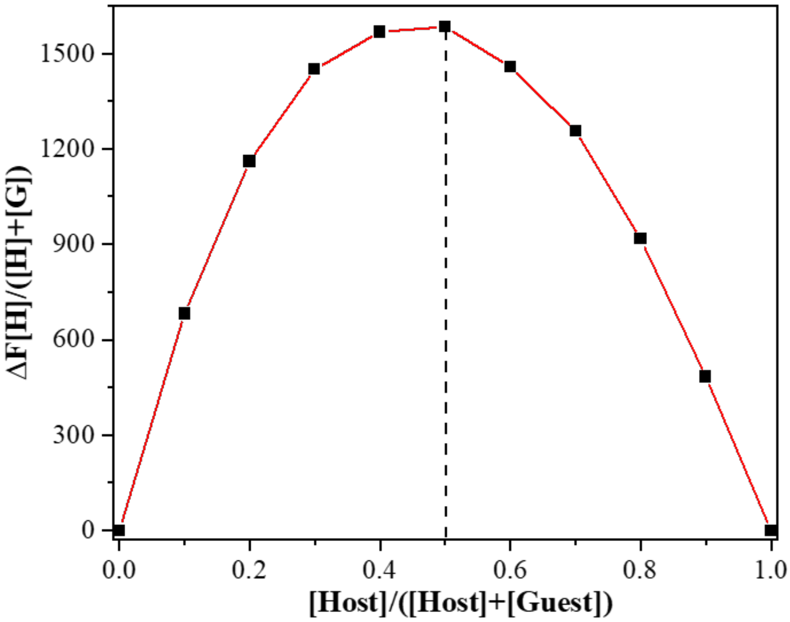

Fig. S14. Job plot for the complexation of compound **1** and C_{60}, derived from fluorescence titration experiments in toluene ([H] + [G] = 1 × 10^{-5} M). The maximum value was found at 0.5, a finding consistent with (but not a proof of) a 1:1 (host:guest) binding stoichiometry.[S9]

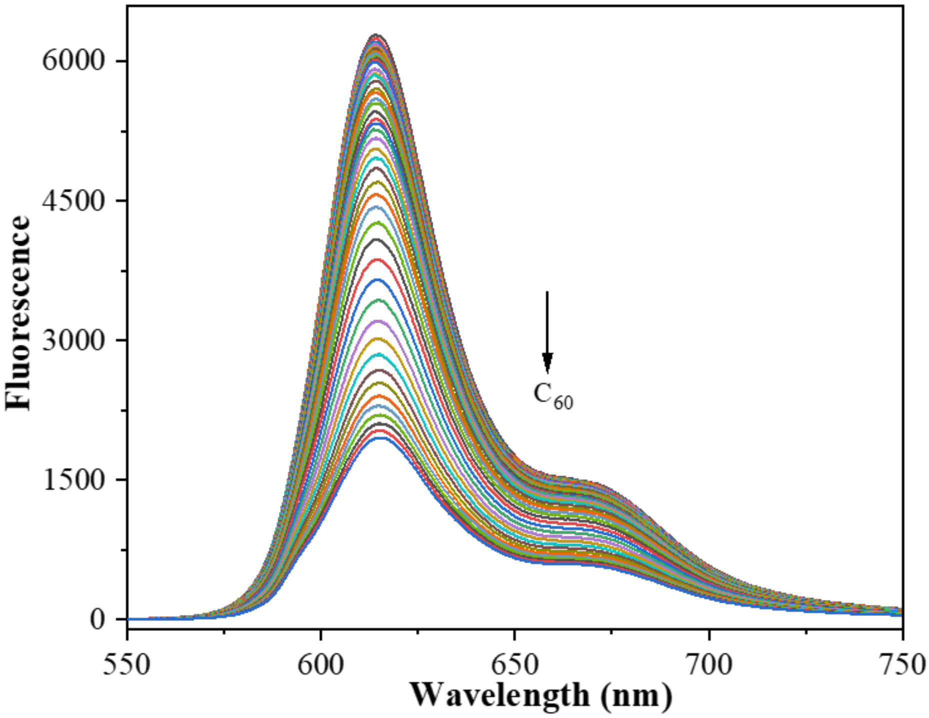

Fig. S15. Fluorescence emission spectra of compound 1 ([**1**] = 1 × 10^{-5} M) in toluene upon the incremental addition of C_{60} (0 - 45 molar equiv.).

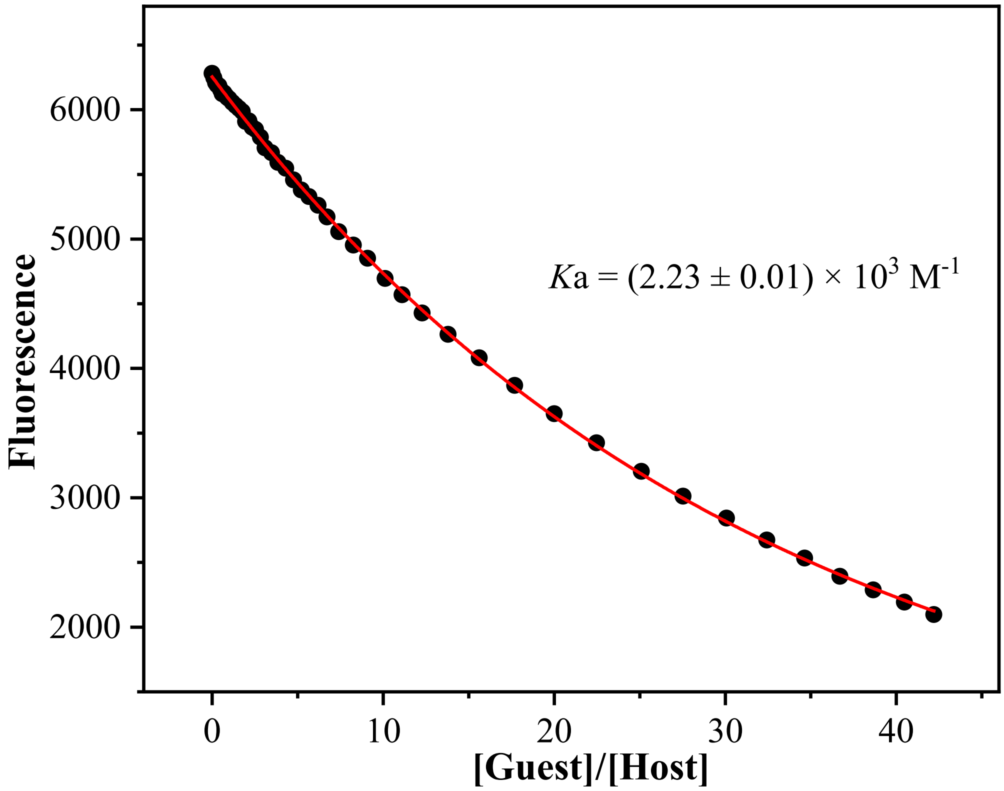

Fig. S16. Binding constant of compound **1** and C_{60}, determined from fluorescence titration measurements in toluene (λ_{em} = 614 nm). Calculation results are accessible at http://app.supramolecular.org/bindfit/view/147a12ba-bf34-40cf-8d0b-7c75eb18f90b.

12. Association behavior of 1 with pyrene

In this study, "H" (Host) refers to compound **1**, while "G" (Guest) denotes pyrene. These abbreviations will be used throughout the text for clarity and consistency.

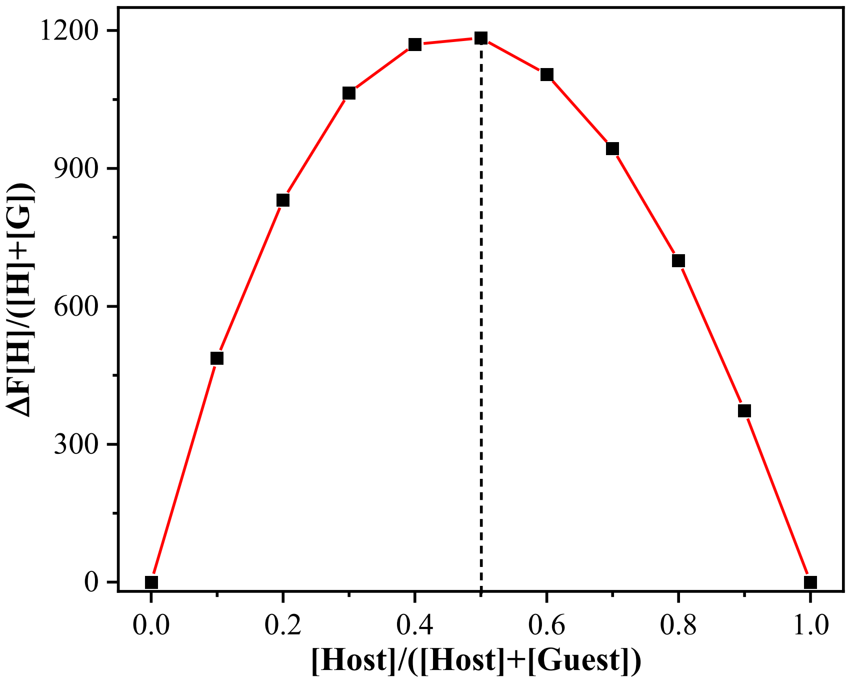

Fig. S17. Job plot for the complexation of compound **1** and pyrene, derived from fluorescence titration experiments in toluene ([H] + [G] = 1 × 10^{-5} M). The maximum value was found at 0.5, a finding consistent with (but not a proof of) a 1:1 (host:guest) binding stoichiometry. [S9]

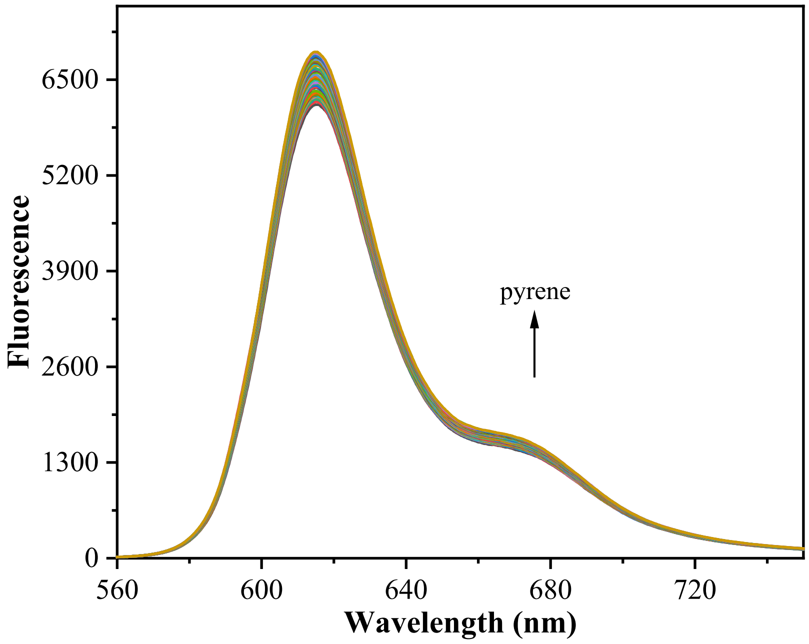

Fig. S18. Fluorescence emission spectra of compound **1** ([**1**] = 1 × 10^{-5} M) in toluene upon the incremental addition of pyrene (0 - 30 molar equiv.).

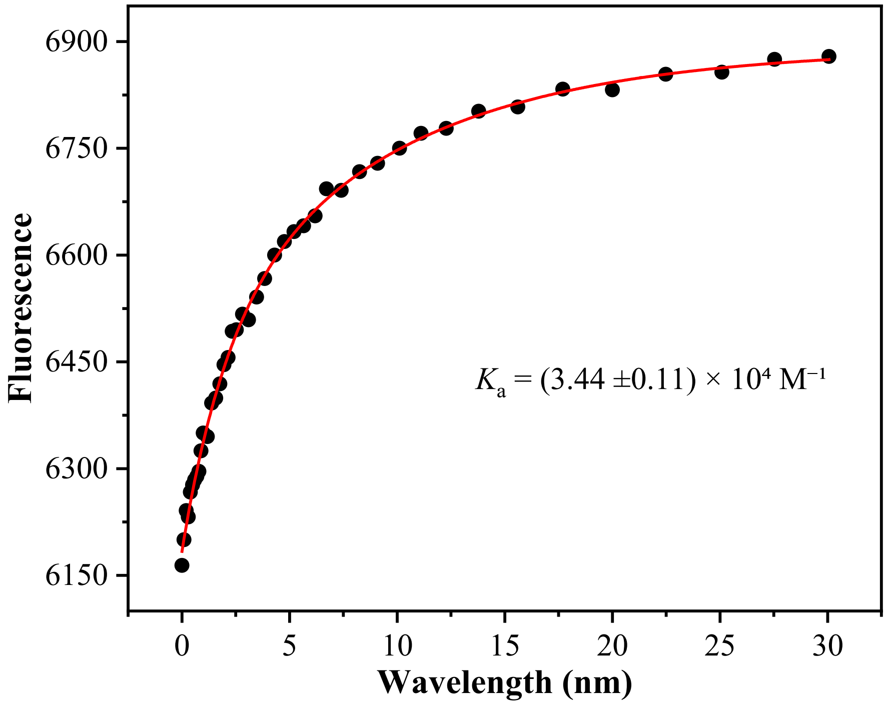

Fig. S19. Binding constant of compound **1** and pyrene, determined from fluorescence titration measurements in toluene (λ_{em} = 614 nm). Calculation results are accessible at http://app.supramolecular.org/bindfit/view/9c85fe25-973e-432d-8ea6-0a2295bb94c8

13. Theoretical calculations on the host-guest complex

All theoretical calculations were conducted using Gaussian 16 software.[S5] Geometry optimizations were carried out at the B3LYP/6-31G(d) level of theory. Independent gradient model based on Hirshfeld partitioning (IGMH) and reduced density gradient (RDG) analyses were performed using Multiwfn, following established protocols from the literature.[S7a,S8]

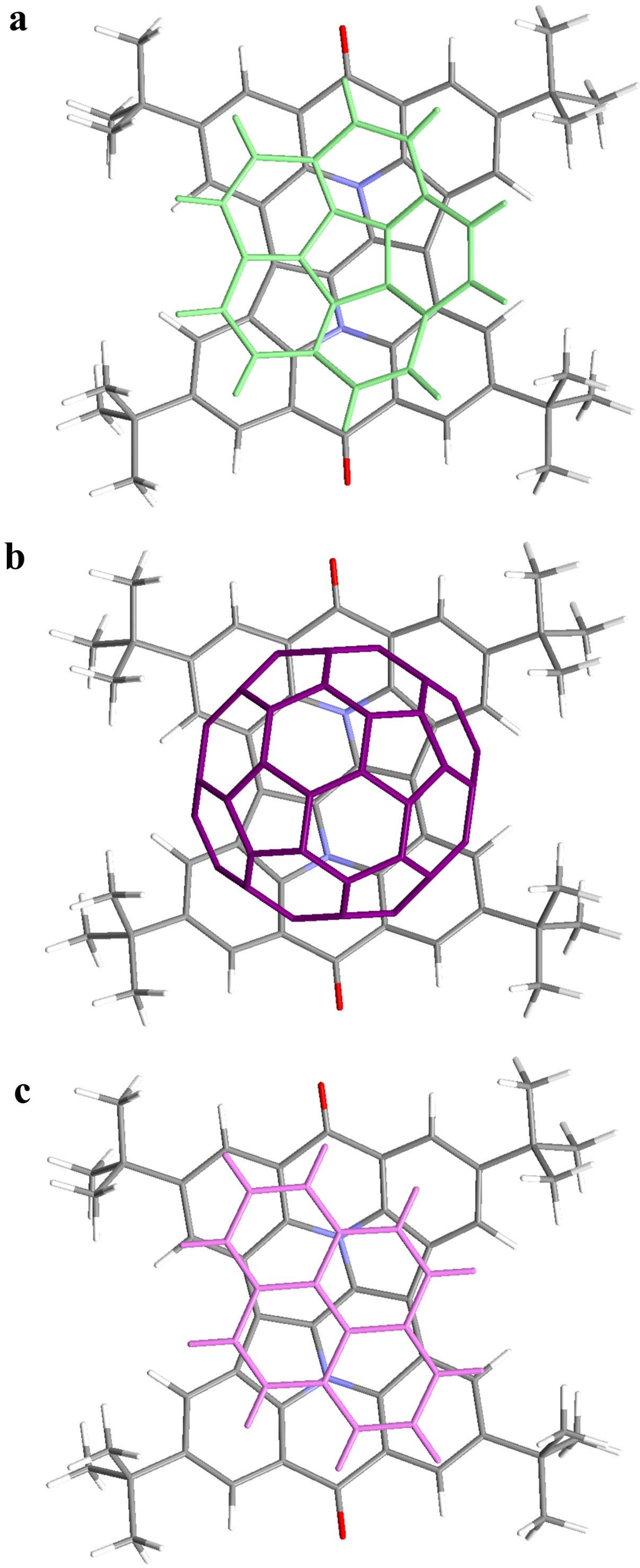

Fig. S20. Superposition of the core structures of compound **1**, corannulene, C_{60}, and pyrene in the optimized geometries of the complexes **1**•corannulene (a), **1**•C_{60} (b), and **1**•pyrene (c).

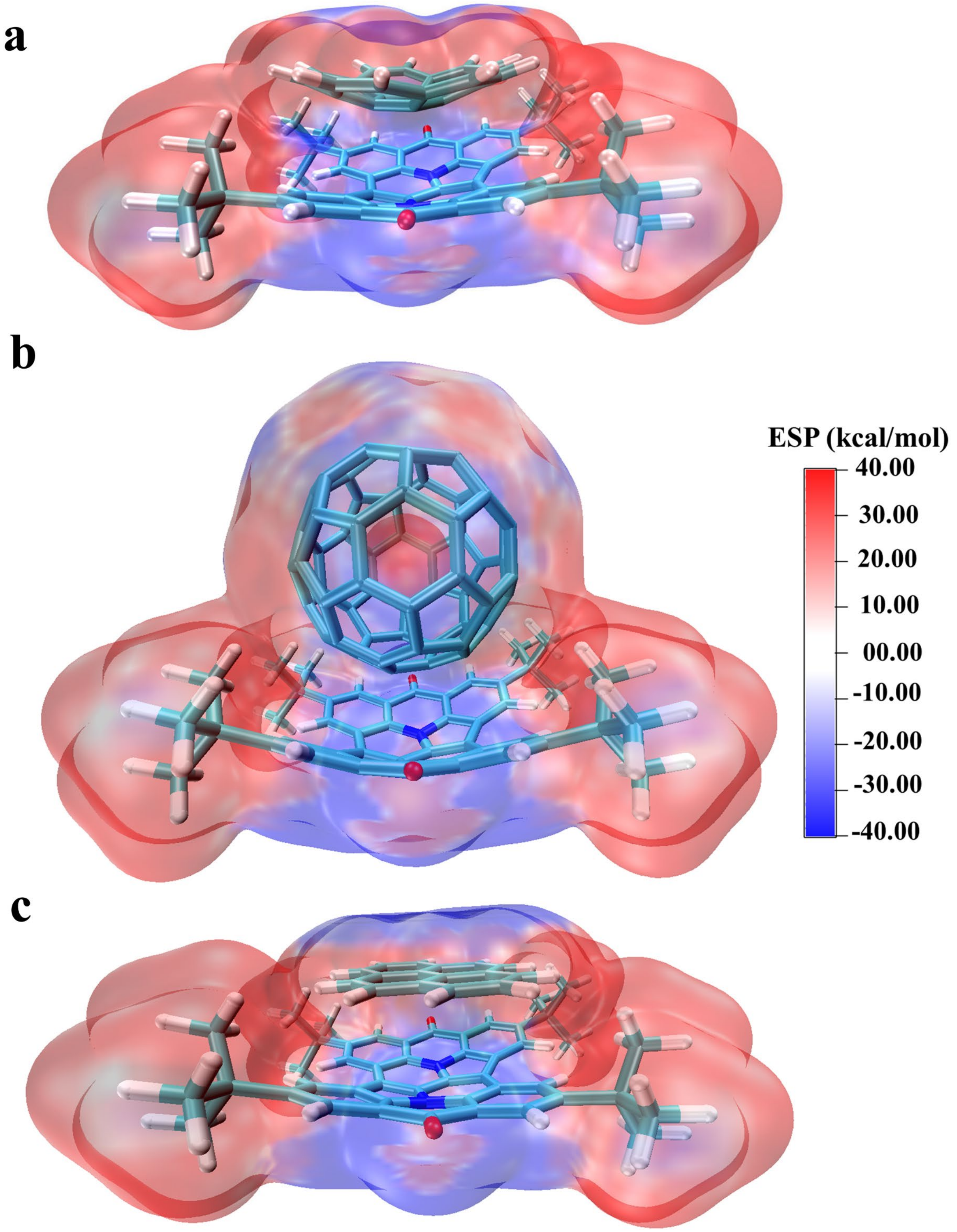

Fig. S21. Electrostatic potential (ESP) diagrams illustrating the charge distributions of the complexes: **1**•corannulene (a), **1**•C_{60} (b), and **1**•pyrene (c).

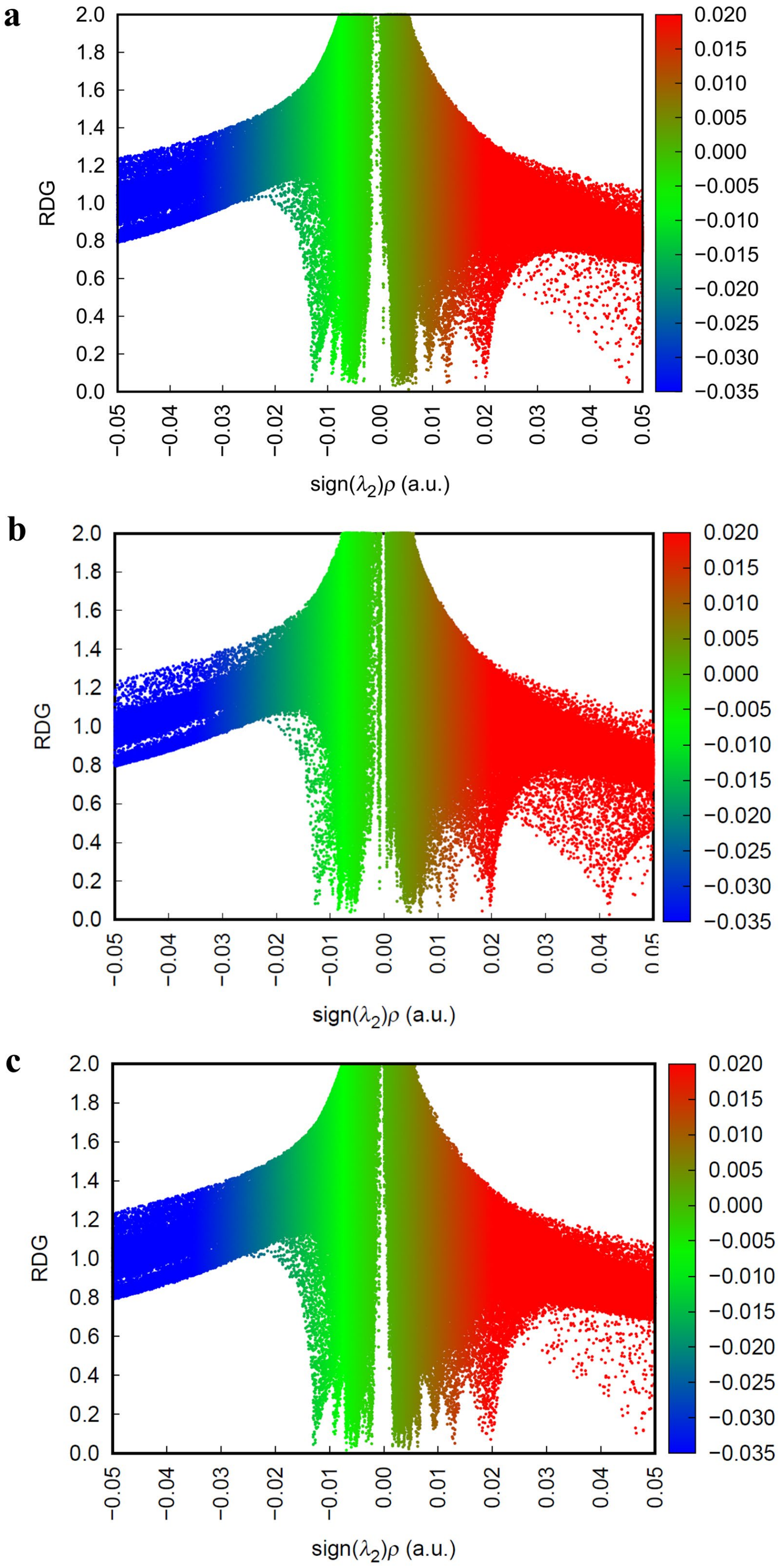

Fig. S22. Reduced density gradient (RDG) analysis highlighting the non-covalent interaction regions in the complexes: **1**•corannulene (a), **1**•C_{60} (b), and **1**•pyrene (c).

14. X-ray crystallographic structure determination

Single crystals of the **1**•corannulene complex were prepared via slow volatilization of a 2 mg/mL solution containing **1** (2 mg) and corannulene (2 mg) in a 1:1 (v/v) mixture of CH_2Cl_2 and hexane at room temperature. After a two-day period, crystals suitable for X-ray diffraction analysis were obtained. The crystallographic data, associated with CCDC number 2412345, are summarized in Table S3.

Table S3. Crystallographic summary of the **1**•corannulene complex.

CCDC No.	2412345
Empirical formula	$C_{66}H_{54}N_2O_2$
Formula weight	907.11
Temperature/K	170(2)
Crystal system	monoclinic
Space group	Pn
a/Å	17.9514(9)
b/Å	7.1173(7)
c/Å	18.5132(9)
α/°	90
β/°	97.763(5)
γ/°	90
Volume/Å^3	2343.7(3)
Z	2
ρ_{calc}g/cm^3	1.285
μ/mm^{-1}	0.591
F(000)	960.0
Crystal size/mm^3	0.2 × 0.15 × 0.05
Radiation	CuKα (λ = 1.54184)
2Θ range for data collection/°	6.438 to 124.934
Index ranges	$-20 \leq h \leq 19$, $-8 \leq k \leq 8$, $-21 \leq l \leq 21$
Reflections collected	24729
Independent reflections	6835 [R_{int} = 0.0979, R_{sigma} = 0.0659]
Data/restraints/parameters	6835/681/496
Goodness-of-fit on F^2	0.957
Final R indexes [I>=2σ (I)]	R_1 = 0.0878, wR_2 = 0.2223
Final R indexes [all data]	R_1 = 0.1263, wR_2 = 0.2509
Largest diff. peak/hole / e Å^{-3}	0.25/-0.21
Flack parameter	0.4(10)

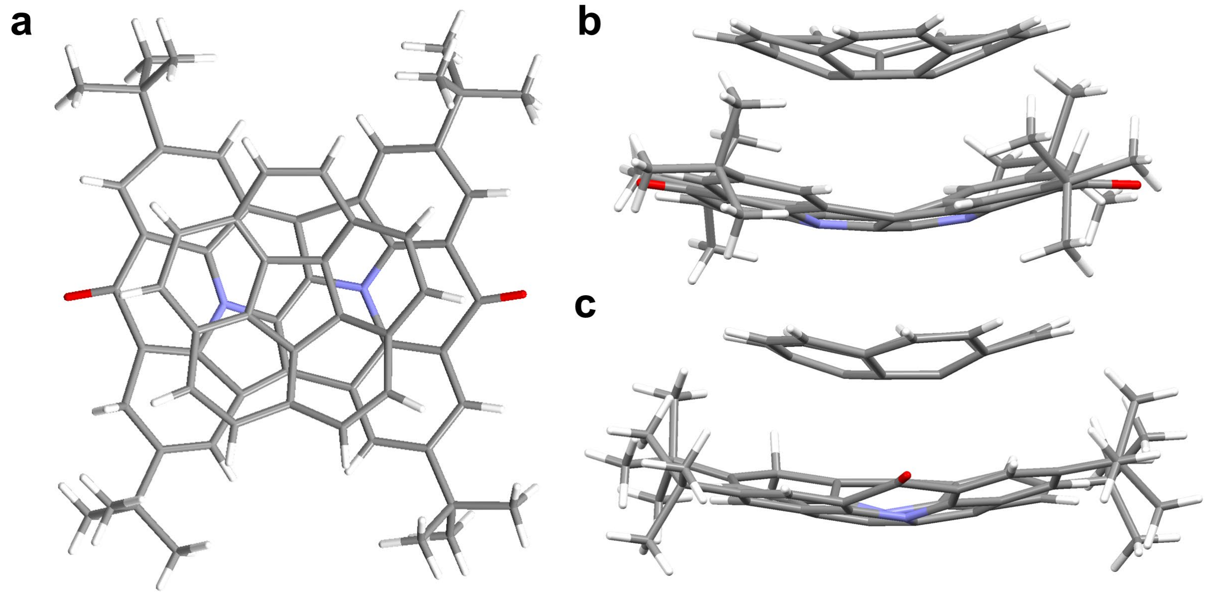

Fig. S23. Three views of the single-crystal structure of the **1**•corannulene complex.

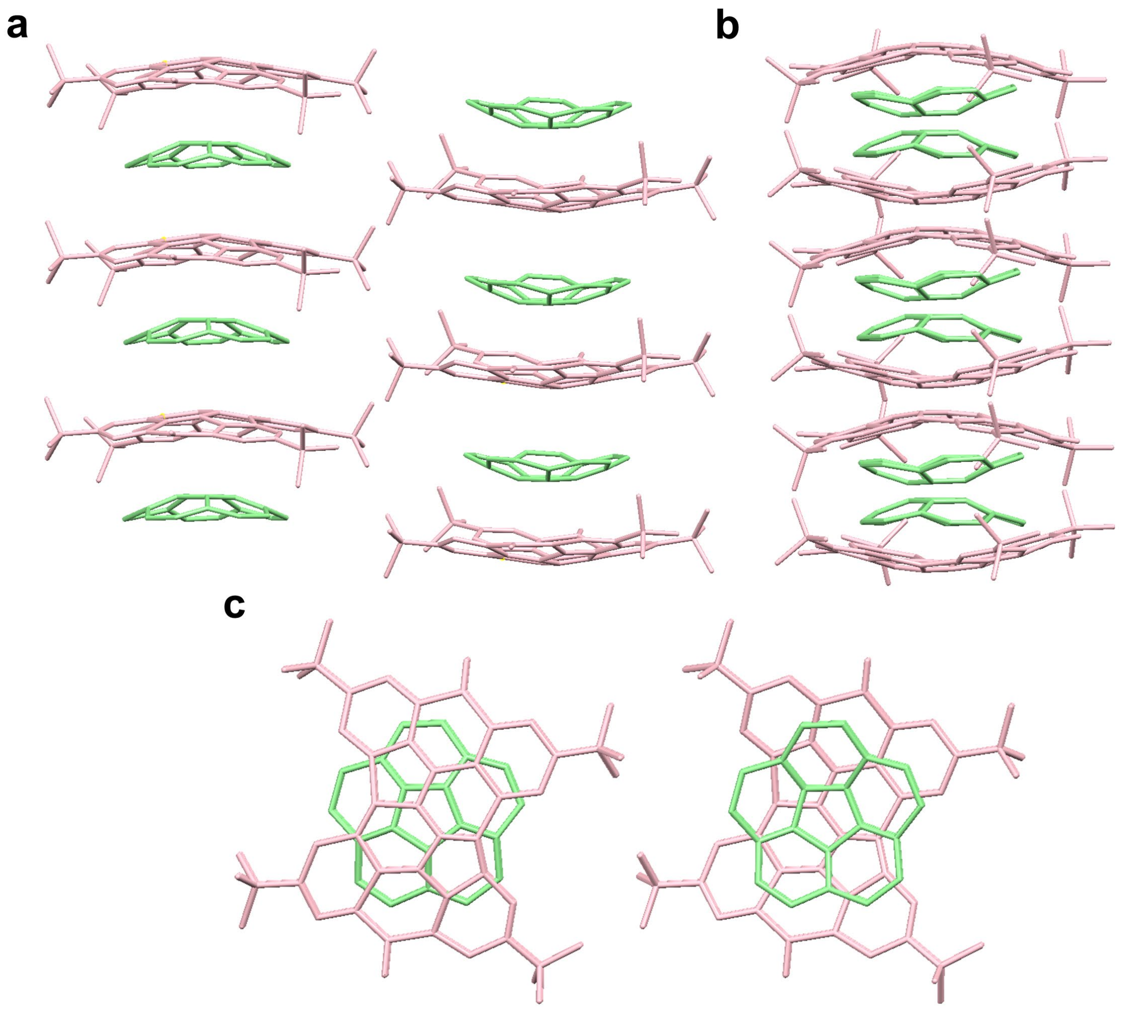

Fig. S24. Three views of the bidirectional columnar packing structure in the single-crystal structure of **1**•corannulene, with corannulene represented in green and compound **1** in pink.

15. Cartesian coordinates for theoretically optimized structures

Table S4. Cartesian coordinates of the theoretically optimized structure of compound **1**.

Compound **1** opt B3LYP/6-31G(d) HF = -2039.453036 Hartree			
C	-4.73952500	1.97230900	0.18199400
C	-4.17950100	0.70448500	-0.09242200
C	-2.81586700	0.56871700	-0.40555400
C	-2.10182700	1.80284900	-0.44281400
C	-2.56831800	3.07031100	-0.13279600
C	-3.93040000	3.13655800	0.17438000
N	-0.79208400	1.56771100	-0.71812000
C	0.26274800	2.43846300	-0.52072700
C	-0.09608400	3.76720700	-0.23899700
C	-1.55207800	4.17372600	-0.08057100
C	1.61287500	1.95175800	-0.52236900
C	2.59560100	2.93013600	-0.30623800
C	2.29050900	4.28616500	-0.07684300
C	0.94323800	4.68049500	-0.03117300
O	-1.86281000	5.33999800	0.13582900
C	-6.23890200	2.13208000	0.51829900
C	3.38929100	5.34562300	0.14539100
C	3.26606200	6.44088000	-0.94079800
C	3.21246800	5.98682300	1.54259100
C	4.80916500	4.75282500	0.06751700
C	-6.39151000	2.73583100	1.93511500
C	-6.89696900	3.07703600	-0.51582900
C	-7.00139200	0.79332600	0.48875300
C	-0.64818600	0.22746200	-0.84571300
C	-1.81800800	-0.49279900	-0.65453000
C	1.81800000	0.49280200	-0.65454500
C	0.64817800	-0.22745800	-0.84572600
C	-2.29050600	-4.28616200	-0.07682000
C	-2.59560400	-2.93013700	-0.30622100
C	-1.61287900	-1.95175400	-0.52235100
C	-0.26275300	-2.43846100	-0.52074800
C	0.09608400	-3.76721600	-0.23906600
C	-0.94323600	-4.68049600	-0.03118900

N	0.79207800	-1.56770800	-0.71813900
C	2.10181700	-1.80284200	-0.44281000
C	2.56831100	-3.07030700	-0.13280900
C	1.55207800	-4.17372900	-0.08062600
C	2.81586400	-0.56871300	-0.40558700
C	4.17949400	-0.70447900	-0.09244000
C	4.73950700	-1.97229500	0.18203300
C	3.93037400	-3.13653900	0.17445600
O	1.86281600	-5.34000900	0.13572800
C	-3.38927100	-5.34563300	0.14541600
C	6.23888700	-2.13206900	0.51832200
C	6.39151300	-2.73575400	1.93516100
C	6.89691900	-3.07708300	-0.51577300
C	7.00139500	-0.79332600	0.48870100
C	-3.26614100	-6.44080300	-0.94086800
C	-3.21230700	-5.98694400	1.54254200
C	-4.80914700	-4.75283900	0.06770900
H	-4.80832800	-0.17651500	-0.04565700
H	-4.35421100	4.10309900	0.42550100
H	3.63089800	2.60947200	-0.30600300
H	0.66470800	5.70717800	0.18196000
H	3.39648300	6.01627700	-1.94276800
H	4.03411200	7.21018900	-0.79491000
H	2.28967200	6.93504200	-0.91183600
H	3.29617600	5.23317800	2.33397500
H	3.98560100	6.74587400	1.71281900
H	2.23848100	6.47531000	1.64787200
H	5.01176800	4.30066400	-0.91028800
H	5.54672400	5.54838600	0.22033600
H	4.98083900	3.99461700	0.84046500
H	-5.94053200	2.08232200	2.69064800
H	-7.45284400	2.85957200	2.18240300
H	-5.91523300	3.71817000	2.01599000
H	-6.81308200	2.66828400	-1.52933400
H	-7.96208500	3.20708600	-0.28870900
H	-6.43352900	4.06884000	-0.51682700
H	-6.95688700	0.31777500	-0.49778100
H	-6.61684700	0.08468000	1.23136200

H	-8.05765900	0.96876600	0.72104100
H	-3.63090100	-2.60947200	-0.30595800
H	-0.66470600	-5.70719300	0.18187700
H	4.80833900	0.17651300	-0.04576100
H	4.35419300	-4.10308500	0.42554600
H	5.94054600	-2.08220900	2.69067100
H	7.45285100	-2.85948400	2.18243800
H	5.91523500	-3.71808900	2.01609200
H	6.81301600	-2.66837900	-1.52929600
H	7.96203800	-3.20713800	-0.28867000
H	6.43346200	-4.06887900	-0.51671200
H	6.95692400	-0.31784200	-0.49786800
H	8.05765300	-0.96876300	0.72102900
H	6.61683600	-0.08462300	1.23124900
H	-3.39668200	-6.01612400	-1.94279000
H	-4.03416000	-7.21013600	-0.79495400
H	-2.28974100	-6.93495400	-0.91205300
H	-3.29588600	-5.23335600	2.33399400
H	-3.98544300	-6.74598300	1.71281000
H	-2.23831800	-6.47546400	1.64765800
H	-5.01184500	-4.30063000	-0.91005300
H	-4.98074900	-3.99467300	0.84071500
H	-5.54668400	-5.54841400	0.22056200

Table S5. Cartesian coordinates of the ground-state structure of compound **1**, calculated for single-point energy.

Unsubstituted compound **1** opt B3LYP/6-311+G(2d,p) HF = -1410.8019131Hartree			
C	3.81631800	-3.42838600	-0.35955100
C	2.42903100	-3.49345700	-0.13333700
C	1.71602600	-2.31341300	0.13175700
C	2.53629000	-1.13904900	0.16000300
C	3.89423000	-1.01858200	-0.09692000
C	4.55111900	-2.22870400	-0.35736500
N	1.75725800	-0.04932500	0.39325800
C	2.10353100	1.27900700	0.22963000
C	3.46987900	1.51591000	-0.00328900
C	4.45888300	0.37052300	-0.13467500

C	1.07920700	2.29354100	0.23485300
C	1.55198600	3.60080300	0.05722700
C	2.91622000	3.86917200	-0.13149200
C	3.86692600	2.85011500	-0.17538400
O	5.65255000	0.58588700	-0.31077100
C	0.48220300	-0.48807100	0.50375000
C	0.32832500	-1.85441800	0.34371900
C	-0.32832400	1.85441800	0.34372100
C	-0.48220200	0.48807100	0.50375100
C	-2.91622000	-3.86917200	-0.13148700
C	-1.55198600	-3.60080300	0.05722900
C	-1.07920600	-2.29354100	0.23485400
C	-2.10353100	-1.27900800	0.22963300
C	-3.46987900	-1.51591000	-0.00328400
C	-3.86692600	-2.85011500	-0.17537700
N	-1.75725700	0.04932500	0.39325900
C	-2.53628900	1.13904900	0.16000700
C	-3.89423000	1.01858200	-0.09691200
C	-4.45888300	-0.37052300	-0.13466900
C	-1.71602600	2.31341300	0.13176200
C	-2.42903100	3.49345800	-0.13332700
C	-3.81632000	3.42838700	-0.35953700
C	-4.55112000	2.22870500	-0.35735200
O	-5.65255100	-0.58588700	-0.31075600
H	1.92879300	-4.45655200	-0.18138600
H	5.61536100	-2.22953400	-0.57002800
H	0.84308100	4.42409300	0.05383900
H	4.91634500	3.06074900	-0.35343200
H	-0.84308100	-4.42409300	0.05384100
H	-4.91634600	-3.06074900	-0.35342300
H	-1.92879400	4.45655200	-0.18137400
H	-5.61536300	2.22953500	-0.57001200
H	-3.23257200	-4.89970400	-0.26440400
H	-4.34334000	4.35582900	-0.56512000
H	3.23257100	4.89970400	-0.26441100
H	4.34333800	-4.35582800	-0.56513700

Table S6. Cartesian coordinates of the transition-state structure of compound **1**, calculated for single-point energy.

Transition intermediate of unsubstituted compound **1** opt B3LYP/6-311+G(2d,p) HF = -1410.8008579 Hartree			
C	3.91008500	3.42829700	-0.00006300
C	2.50391600	3.50158100	-0.00002900
C	1.74950500	2.31732800	-0.00003400
C	2.55744000	1.13085300	-0.00007700
C	3.93827400	1.00353800	-0.00010600
C	4.63315000	2.22062900	-0.00009900
N	1.75145200	0.04025500	-0.00007000
C	2.10167000	-1.29356200	-0.00007800
C	3.48622900	-1.53770900	-0.00011200
C	4.49002900	-0.39382400	-0.00012900
C	1.07227700	-2.30705700	-0.00005200
C	1.55982200	-3.62099400	-0.00006700
C	2.93672800	-3.89473700	-0.00010400
C	3.89369200	-2.87970000	-0.00012500
O	5.69502900	-0.61778800	-0.00015700
C	0.48062400	0.48569200	-0.00001500
C	0.34077100	1.86095400	0.00000600
C	-0.34077100	-1.86095400	-0.00000500
C	-0.48062400	-0.48569200	0.00001600
C	-2.93672800	3.89473700	0.00010500
C	-1.55982200	3.62099400	0.00006800
C	-1.07227700	2.30705700	0.00005200
C	-2.10167000	1.29356200	0.00007600
C	-3.48622900	1.53770900	0.00011100
C	-3.89369200	2.87970000	0.00012500
N	-1.75145200	-0.04025500	0.00006500
C	-2.55744000	-1.13085300	0.00007000
C	-3.93827400	-1.00353800	0.00010300
C	-4.49002900	0.39382400	0.00012800
C	-1.74950500	-2.31732800	0.00003100
C	-2.50391600	-3.50158100	0.00003300
C	-3.91008500	-3.42829700	0.00006800
C	-4.63315000	-2.22062900	0.00010200
O	-5.69502900	0.61778800	0.00015900

H	2.02508500	4.47672200	0.00000300
H	5.71840900	2.22474200	-0.00011900
H	0.85479000	-4.44767200	-0.00004800
H	4.95566500	-3.10225500	-0.00015000
H	-0.85479000	4.44767200	0.00005000
H	-4.95566500	3.10225500	0.00015200
H	-2.02508500	-4.47672200	0.00000600
H	-5.71840900	-2.22474200	0.00012500
H	-3.26135000	4.93128700	0.00011600
H	-4.46705500	-4.36103900	0.00006800
H	3.26135000	-4.93128700	-0.00011500
H	4.46705500	4.36103900	-0.00005800

Table S7. Cartesian coordinates of the theoretically optimized structure of the **1**•corannulene complex.

Complex of **1**-corannulene opt B3LYP/6-31G(d) em=gd3bj HF = -2807.9852627 Hartree			
O	-1.12390800	-5.48955500	-0.44291900
O	1.28398600	5.38858200	-0.52808900
C	-4.34971300	-2.50107900	-0.67062900
C	-3.42475000	-3.56278300	-0.62045500
H	-3.72055000	-4.54533200	-0.27704500
C	-2.08196200	-3.36785800	-0.97280700
C	-3.93735400	-1.19816900	-1.04408900
H	-4.65633400	-0.38647900	-1.01113400
C	-0.94945300	-4.32978200	-0.80658100
C	0.44409100	-3.77705500	-1.01491400
C	0.64927200	-2.44822600	-1.40729000
C	1.93316000	-1.80267500	-1.39836900
C	3.01019000	-2.62839400	-1.07216800
H	3.99991500	-2.18359100	-1.04984500
C	2.86084100	-3.99319100	-0.74539900
C	1.57777600	-4.54244300	-0.70077300
H	1.40809600	-5.56862900	-0.40138500
C	-0.49636700	-0.37427000	-1.88791600
N	-0.49003900	-1.72239300	-1.70003200
C	-1.76563100	-2.09152800	-1.39572100
C	-2.61434100	-0.94148900	-1.41064100

C	-1.74180100	0.20944200	-1.71203100
C	-1.71723100	1.67357000	-1.55424100
C	-0.43439400	2.31881300	-1.50302400
C	-0.25083800	3.65605800	-1.12952700
C	-1.39980400	4.42510300	-0.89180200
H	-1.24659000	5.45626500	-0.60158600
C	-2.67869400	3.87347000	-0.99318700
C	-2.81076000	2.50307500	-1.30370700
H	-3.79970100	2.05667300	-1.32095800
C	1.12906400	4.21901700	-0.86789400
C	2.27140900	3.25724500	-0.95401400
C	2.82524600	0.81850800	-1.28349800
C	1.97022500	-0.34301200	-1.59718700
C	0.73689100	0.23389400	-1.86061900
N	0.71941300	1.58615500	-1.70955400
C	1.97645000	1.96645500	-1.34753400
C	4.13395200	1.09506900	-0.87758800
H	4.85593200	0.28923900	-0.79521400
C	4.53006800	2.41261700	-0.54104700
C	3.59924300	3.47044700	-0.55719200
H	3.87977200	4.46652000	-0.24060200
C	-5.82680000	-2.70721800	-0.28969500
C	-6.14033100	-4.16817600	0.07297900
H	-5.92862900	-4.84745400	-0.75961600
H	-7.20296300	-4.26441700	0.32048900
H	-5.56591700	-4.50345000	0.94329300
C	-6.72145500	-2.30821000	-1.48368100
H	-6.48919400	-2.91854400	-2.36316100
H	-6.58733000	-1.25770800	-1.76071900
H	-7.77872800	-2.45552200	-1.23356000
C	-6.17633000	-1.82832300	0.93047200
H	-5.58073200	-2.12409900	1.80048200
H	-7.23613700	-1.93909400	1.18863500
H	-5.98428000	-0.76761500	0.74199700
C	-3.94854500	4.69912500	-0.73861900
C	-3.63302300	6.17895800	-0.46710200
H	-3.01760000	6.30432400	0.43015900
H	-4.56598500	6.73076000	-0.30944900

H	-3.10783100	6.64206000	-1.30927400
C	-4.69112200	4.13275500	0.49039100
H	-4.94026200	3.07408200	0.36767300
H	-5.62357500	4.68413100	0.66000800
H	-4.07063000	4.22401600	1.38760700
C	-4.86723500	4.62126400	-1.97721800
H	-4.35759000	5.01320500	-2.86412600
H	-5.77549500	5.21319400	-1.81421000
H	-5.17428200	3.59318800	-2.19446600
C	5.99508500	2.64243200	-0.12741300
C	6.92374500	2.20463700	-1.28129000
H	6.80303100	1.14360800	-1.52210800
H	7.97311900	2.36829100	-1.00901100
H	6.71159600	2.77984700	-2.18904000
C	6.31906800	1.81398800	1.13445600
H	5.69691600	2.13461700	1.97665900
H	7.37012500	1.94589900	1.41701900
H	6.14638400	0.74434600	0.97788000
C	6.29085800	4.11835800	0.18675900
H	6.09638700	4.76376200	-0.67638400
H	7.34633700	4.23110100	0.45681400
H	5.69306400	4.48296700	1.02908700
C	4.11603700	-4.81481500	-0.41583300
C	3.78307600	-6.28296300	-0.10486400
H	3.13223100	-6.37476400	0.77129400
H	4.70641400	-6.83227700	0.10822700
H	3.28924200	-6.77368500	-0.95026100
C	5.07706200	-4.78327100	-1.62417200
H	4.59717400	-5.20761900	-2.51271600
H	5.97872300	-5.36863700	-1.40887700
H	5.39143800	-3.76345900	-1.86859400
C	4.82167300	-4.21058600	0.81741200
H	5.10124300	-3.16429000	0.65662900
H	5.73560000	-4.77138300	1.04578100
H	4.16721500	-4.25070800	1.69436400
C	0.17112900	3.38432400	2.41721400
H	0.61839800	4.31771700	2.74807600
C	-1.21192900	3.26166100	2.45251000

H	-1.79486300	4.10214800	2.82104600
C	-1.88323600	2.02257900	2.12280000
C	-1.05822300	1.05958600	1.56888200
C	0.35544400	1.18419000	1.53040700
C	1.02793700	2.27790700	2.04866600
C	2.41636800	2.02593700	2.36653300
H	3.04069700	2.84677900	2.70929100
C	2.95522500	0.74634900	2.34599600
H	3.97982200	0.61219000	2.68125300
C	2.16724200	-0.41544900	1.99918800
C	0.90937500	-0.12231800	1.50233700
C	-0.16108200	-1.05474000	1.51794000
C	-0.03482200	-2.33810600	2.02246500
C	1.32480200	-2.72586500	2.32874700
H	1.52678900	-3.74237300	2.65591200
C	2.36945300	-1.81096800	2.32092900
H	3.35007300	-2.14403800	2.65010700
C	-1.28763600	-2.95768200	2.39532600
H	-1.29341000	-3.99480400	2.71978100
C	-2.47611500	-2.24041200	2.44290400
H	-3.36584200	-2.74224000	2.81411900
C	-2.53915900	-0.83218900	2.11804000
C	-1.37698100	-0.32391300	1.56363000
C	-3.50007100	0.17770300	2.50803400
H	-4.46567700	-0.12983100	2.90001800
C	-3.18888700	1.53167200	2.51018200
H	-3.92414600	2.22824300	2.90401900

Table S8. Cartesian coordinates of the theoretically optimized structure of the **1**•C_{60} complex.

Complex of **1**-C_{60} opt B3LYP/6-31G(d) em=gd3bj HF=-4326.2514232 Hartree			
O	2.37427800	0.63517700	-5.42535300
O	2.32128300	-0.78968500	5.42544600
C	2.49929300	4.04014800	-2.66452700
C	2.48428000	3.04473600	-3.66154000
H	2.02658800	3.22652600	-4.62508000
C	2.99919100	1.76375100	-3.41735600

C	3.01414400	3.75310300	-1.37917900
H	2.95739700	4.51180900	-0.60658600
C	2.80870700	0.56093800	-4.28090700
C	3.04942000	-0.77343200	-3.61546700
C	3.56708500	-0.86027100	-2.31571900
C	3.55793600	-2.07571300	-1.55748000
C	3.10354400	-3.21192100	-2.22897500
H	3.08054600	-4.14731600	-1.68058300
C	2.62284400	-3.17898500	-3.55264200
C	2.59243900	-1.95442600	-4.22230900
H	2.17917500	-1.86091000	-5.21824300
C	4.17015400	0.45102200	-0.36341900
N	3.94573400	0.33349800	-1.71300500
C	3.54998900	1.56615600	-2.16334300
C	3.53816200	2.49232200	-1.07950100
C	3.92861900	1.73211000	0.11554100
C	3.68582400	1.83709300	1.55915600
C	3.61391900	0.62377300	2.31728700
C	3.09097400	0.57126100	3.61684000
C	2.71330900	1.77990600	4.22366200
H	2.29419400	1.71407200	5.21936500
C	2.82584300	2.99991300	3.55429800
C	3.30811400	3.00099200	2.23083600
H	3.34884100	3.93605800	1.68294400
C	2.76120800	-0.74431000	4.28158800
C	2.87263500	-1.95708400	3.41809000
C	3.36530900	-2.71971300	1.08084400
C	3.80616300	-1.98696700	-0.11371000
C	4.13093600	-0.72442500	0.36548200
N	3.91331100	-0.59238500	1.71482600
C	3.43653800	-1.79622000	2.16464100
C	2.75950600	-3.94345500	1.38004400
H	2.65436900	-4.69706600	0.60753300
C	2.22501400	-4.19573300	2.66461600
C	2.27411900	-3.20128900	3.66157500
H	1.80403200	-3.35231600	4.62444200
C	1.93982300	5.45042100	-2.92361200
C	1.32758400	5.58402600	-4.32757000

H	2.06745600	5.40484600	-5.11489800
H	0.93913300	6.59905900	-4.46227700
H	0.49488600	4.88684600	-4.47121300
C	3.09282600	6.46990700	-2.79391000
H	3.88778900	6.25244100	-3.51559700
H	3.53582400	6.45264400	-1.79248400
H	2.72669600	7.48600000	-2.98219200
C	0.83727300	5.79015700	-1.89633600
H	-0.01632000	5.11508500	-2.00320400
H	0.48269800	6.81501300	-2.05531900
H	1.19208700	5.71972000	-0.86347900
C	2.39406700	4.33171400	4.18343600
C	1.82929100	4.14375600	5.60045800
H	0.94283100	3.50031500	5.59888900
H	1.53486600	5.11563000	6.01049800
H	2.56990300	3.70643500	6.27854400
C	1.29602500	4.98249900	3.31276300
H	1.63717000	5.16984200	2.28930200
H	0.99468300	5.94450000	3.74322600
H	0.41126400	4.34127500	3.25739500
C	3.61563600	5.27245000	4.26191500
H	4.40642700	4.83324600	4.87981800
H	3.32827500	6.23325500	4.70463200
H	4.03701800	5.47296400	3.27119700
C	1.57179100	-5.56534200	2.92239300
C	2.65373600	-6.66012200	2.79445300
H	3.09806000	-6.67319800	1.79357500
H	2.21985000	-7.64919900	2.98266900
H	3.46066300	-6.49635700	3.51704400
C	0.45067800	-5.82960000	1.89299300
H	-0.35557000	-5.09845600	1.99863700
H	0.02737200	-6.82818800	2.05095200
H	0.81135300	-5.78304300	0.86080400
C	0.94947200	-5.65768200	4.32525200
H	1.69835700	-5.52911300	5.11395500
H	0.49310000	-6.64419400	4.45887600
H	0.16552000	-4.90590600	4.46774900
C	2.10162500	-4.47878800	-4.18079200

C	1.54981300	-4.25385800	-5.59755800
H	0.70856500	-3.55232400	-5.59569600
H	1.19045500	-5.20392700	-6.00693300
H	2.31768500	-3.86762900	-6.27635200
C	3.25634500	-5.50042100	-4.25934300
H	4.07419600	-5.11691300	-4.87910000
H	2.90373700	-6.44021500	-4.70000200
H	3.66462700	-5.72745100	-3.26884900
C	0.96236500	-5.05231400	-3.30881400
H	1.29131400	-5.26415000	-2.28616400
H	0.59354700	-5.99035100	-3.73962800
H	0.12506200	-4.35047200	-3.25122100
C	-3.38282400	-2.82055900	-1.87460200
C	-1.92888800	-2.86512600	-1.88191300
C	-1.20654200	-1.91622600	-2.60771800
C	-1.90595300	-0.88339200	-3.35877300
C	-3.29988800	-0.84138000	-3.35025700
C	-4.05314700	-1.82961000	-2.59268100
C	-1.48355100	-3.26062000	-0.55310400
C	-0.01054100	-1.32086000	-2.03365900
C	-1.13986700	0.35000900	-3.24977800
C	-3.98849400	0.43540900	-3.23411800
C	-5.20773200	-1.16402100	-2.00963600
C	-3.83712500	-3.18677500	-0.54134400
C	-3.25445700	1.61727600	-3.12933900
C	-1.79989900	1.57467700	-3.13829800
C	-3.66932600	2.65041200	-2.19201000
C	-1.31614200	2.58057800	-2.20452900
C	-0.33562000	-2.68848200	-0.00422900
C	0.02824300	0.07887600	-2.42825200
C	-2.47164500	3.24631100	-1.61956300
C	0.48883100	1.04247800	-1.53264600
C	0.41456100	-1.69808300	-0.76137900
C	-5.16737100	0.23568600	-2.40549600
C	-5.64384200	-1.51550400	-0.73224600
C	-0.31980700	-2.28972300	1.39718800
C	-4.94410200	-2.54757000	0.01744300
C	-2.66408500	-3.45876500	0.27592400

C	-6.05880100	-0.48243200	0.20449300
C	-4.92625500	-2.15226400	1.41736000
C	-2.64703100	-3.07808700	1.61855400
C	-0.19381100	2.31995700	-1.41855200
C	-5.56541500	1.22589200	-1.50747600
C	0.89280600	-0.68960200	0.17265700
C	-5.61508900	-0.87611000	1.53303800
C	-3.80177400	-2.41170700	2.20131300
C	-1.44865900	-2.48288800	2.19316900
C	-1.86308300	-1.45020800	3.13101000
C	0.43921200	-1.05559800	1.50510300
C	0.93084700	0.64960900	-0.20408400
C	0.52182700	1.68448100	0.73374600
C	-3.31762000	-1.40554100	3.13473300
C	-3.97861400	-0.18187900	3.24552600
C	-3.21383500	1.05131700	3.35545700
C	-5.15067700	0.08827200	2.42728600
C	-4.80033900	2.45838400	-1.39808000
C	0.08634900	1.33317000	2.00986900
C	-6.02046900	0.85913600	-0.17498600
C	-5.53629100	1.86533400	0.75782800
C	0.04485400	-0.06639200	2.40443200
C	-0.17390000	2.71834100	-0.01696900
C	-1.28044200	3.35827700	0.54184300
C	-1.12989200	-0.26732800	3.23664800
C	-1.81985000	1.00976000	3.35189400
C	-5.11064000	1.48797900	2.03138400
C	-3.91312000	2.08304200	2.60412900
C	-1.06646100	1.99906300	2.59442800
C	-4.78247300	2.85382900	0.00175100
C	-2.45420700	3.62687000	-0.27691500
C	-1.73693900	2.98984300	1.87469400
C	-3.63421100	3.42521900	0.55063500
C	-3.19093700	3.03206300	1.87987200

Table S9. Cartesian coordinates of the theoretically optimized structure of the **1**•pyrene complex.

Complex of **1**- pyrene opt B3LYP/6-31G(d) em=gd3bj HF=-4326.2514232 Hartree			
O	-1.93459	-5.08351	-0.64974
O	2.38171	5.28388	-0.35022
C	-4.60913	-1.57712	-0.71743
C	-3.87825	-2.77986	-0.70805
H	-4.35791	-3.72110	-0.48486
C	-2.50241	-2.78912	-0.96441
C	-3.96524	-0.33791	-0.94106
H	-4.54618	0.57415	-0.89820
C	-1.55714	-3.94554	-0.88033
C	-0.08001	-3.62397	-1.01413
C	0.35749	-2.32805	-1.29805
C	1.73264	-1.91833	-1.26744
C	2.64640	-2.93797	-1.01195
H	3.69739	-2.67964	-0.97759
C	2.25641	-4.26976	-0.76264
C	0.89943	-4.59267	-0.75775
H	0.55386	-5.59222	-0.53728
C	-0.40633	-0.06415	-1.62950
N	-0.63372	-1.39384	-1.50405
C	-1.95644	-1.55579	-1.24354
C	-2.59623	-0.27973	-1.20646
C	-1.52978	0.71863	-1.43039
C	-1.24239	2.15256	-1.26006
C	0.13298	2.56671	-1.25401
C	0.55455	3.85326	-0.90869
C	-0.43338	4.81254	-0.64962
H	-0.09556	5.80315	-0.38209
C	-1.78777	4.48623	-0.69408
C	-2.16447	3.15914	-0.98205
H	-3.21317	2.89091	-0.96450
C	2.01718	4.16292	-0.66925
C	2.96281	3.00380	-0.72704
C	3.06868	0.50778	-1.06141
C	2.02118	-0.47882	-1.38630

C	0.90993	0.31381	-1.61693
N	1.13654	1.64317	-1.47208
C	2.44462	1.79225	-1.13008
C	4.38335	0.53451	-0.59460
H	4.93798	-0.38939	-0.49140
C	4.99514	1.75145	-0.21017
C	4.28663	2.96584	-0.2716
H	4.73310	3.88967	0.06559
C	-6.13275	-1.57097	-0.49565
C	-6.68605	-2.97381	-0.19990
H	-6.50875	-3.66264	-1.02892
H	-7.76577	-2.91374	-0.04354
H	-6.24241	-3.40217	0.70249
C	-6.81760	-1.04246	-1.77475
H	-6.56970	-1.67252	-2.63239
H	-6.50318	-0.02259	-2.00724
H	-7.90459	-1.04048	-1.65182
C	-6.50160	-0.65867	0.69192
H	-6.06256	-1.03138	1.61981
H	-7.58728	-0.63019	0.82047
H	-6.15800	0.36699	0.54477
C	-2.88813	5.51810	-0.40697
C	-2.31276	6.91133	-0.11113
H	-1.67161	6.90543	0.77370
H	-3.13022	7.61186	0.07572
H	-1.73069	7.29414	-0.95271
C	-3.71079	5.06651	0.81823
H	-4.17441	4.09054	0.65940
H	-4.50790	5.78589	1.02551
H	-3.07662	4.99812	1.70584
C	-3.81521	5.62796	-1.63590
H	-3.25133	5.94343	-2.51711
H	-4.60356	6.36330	-1.45145
H	-4.29427	4.67449	-1.86867
C	6.43985	1.69383	0.31605
C	7.35959	1.10483	-0.77436
H	7.06025	0.09298	-1.05578
H	8.39181	1.06059	-0.41553

H	7.33603	1.72373	-1.67467
C	6.49047	0.79897	1.57320
H	5.84089	1.19653	2.35728
H	7.51031	0.75355	1.96548
H	6.17103	-0.22314	1.35868
C	6.97916	3.08135	0.69689
H	6.98281	3.76232	-0.15758
H	8.00829	2.9864	1.05136
H	6.39400	3.53858	1.49843
C	3.34563	-5.31133	-0.46821
C	2.75908	-6.70963	-0.22225
H	2.09108	-6.72241	0.64243
H	3.56929	-7.41599	-0.02608
H	2.20269	-7.07205	-1.08985
C	4.31081	-5.39614	-1.66931
H	3.77472	-5.69143	-2.57454
H	5.09186	-6.13692	-1.47591
H	4.79893	-4.43926	-1.86639
C	4.12895	-4.88845	0.79313
H	4.60392	-3.91285	0.66883
H	4.91429	-5.61677	1.01395
H	3.46597	-4.8312	1.66040
C	0.29230	2.51232	2.03722
C	-1.06415	2.80173	2.13095
C	-2.00499	1.77951	2.18800
C	-1.60578	0.43835	2.17176
C	-2.54413	-0.64435	2.25098
C	-2.12914	-1.9357	2.30512
C	-0.73478	-2.27489	2.27934
C	-0.27867	-3.59398	2.38971
C	1.08157	-3.8818	2.36858
C	2.01663	-2.86384	2.23184
C	1.61029	-1.52653	2.13026
C	2.54657	-0.4469	2.01134
C	2.13196	0.84445	1.95808
C	0.74030	1.18468	2.02290
C	-0.21613	0.13132	2.10184
C	0.21864	-1.22343	2.15583

H	1.01696	3.31532	1.97441
H	-1.39312	3.83361	2.14352
H	-3.06062	2.01647	2.23636
H	-3.60051	-0.40438	2.28328
H	-2.85075	-2.73985	2.37955
H	-1.00140	-4.39511	2.48655
H	1.41705	-4.90967	2.43275
H	3.07369	-3.09892	2.19378
H	3.60088	-0.68814	1.95423
H	2.85113	1.65005	1.86383

16. References

S1 G. Zhu and G. Zhang, *Org. Chem. Front.*, 2021, **8**, 5336–5344.

S2 P. Thordarson, *Chem. Soc. Rev.*, 2011, **40**, 1305–1323.

S3 D. B. Hibbert and P. Thordarson, *Chem. Commun.***,** 2016, **52**, 12792-12805.

S4 G. M. Sheldrick, *Acta Cryst.*, 2015, **71**, 3–8.

S5 M. J. Frisch, G. W. Trucks, H. B. Schlegel, G. E. Scuseria, M. A. Robb, J. R. Cheeseman, G. Scalmani, V. Barone, G. A. Petersson, H. Nakatsuji, X. Li, M. Caricato, A. V. Marenich, J. Bloino, B. G. Janesko, R. Gomperts, B. Mennucci, H. P. Hratchian, J. V. Ortiz, A. F. Izmaylov, J. L. Sonnenberg, D. Williams-Young, F. Ding, F. Lipparini, F. Egidi, J. Goings, B. Peng, A. Petrone, T. Henderson, D. Ranasinghe, V. G. Zakrzewski, J. Gao, N. Rega, G. Zheng, W. Liang, M. Hada, M. Ehara, K. Toyota, R. Fukuda, J. Hasegawa, M. Ishida, T. Nakajima, Y. Honda, O. Kitao, H. Nakai, T. Vreven, K. Throssell, J. A. Montgomery, Jr., J. E. Peralta, F. Ogliaro, M. J. Bearpark, J. J. Heyd, E. N. Brothers, K. N. Kudin, V. N. Staroverov, T. A. Keith, R. Kobayashi, J. Normand, K. Raghavachari, A. P. Rendell, J. C. Burant, S. S. Iyengar, J. Tomasi, M. Cossi, J. M. Millam, M. Klene, C. Adamo, R. Cammi, J. W. Ochterski, R. L. Martin, K. Morokuma, O. Farkas, J. B. Foresman and D. J. Fox, Gaussian 16, Revision A.03, Gaussian, Inc., Wallingford CT, 2016.

S6 T. Amaya, H. Sakane, T. Nakata and T. Hirao, *Pure Appl. Chem.*, 2010, **82**, 969–978.

S7 (a) T. Lu and F. Chen, *J. Comput. Chem.*, 2012, **33**, 580–592; (b) H. Fallah-Bagher-Shaidaei, C. S. Wannere, C. Corminboeuf, R. Puchta and P. v. R. Schleyer, *Org. Lett.*, 2006, **8**, 863–866; (c) S. Klod and E. Kleinpeter, *J. Chem. Soc. Perkin Trans. 2*, 2001, 1893–1898; (d) Z. Liu, T. Lu and Q. Chen, *Carbon*, 2020, **165**, 468–475.

S8 T. Lu and Q. Chen, *J. Comput. Chem.*, 2022, **43**, 539–555.

S9 P. Job, *Ann. Chim. Paris.*, 1928, **9**, 113–203.
